# Smoking‐Induced M2‐TAMs, via circEML4 in EVs, Promote the Progression of NSCLC through ALKBH5‐Regulated m6A Modification of SOCS2 in NSCLC Cells

**DOI:** 10.1002/advs.202300953

**Published:** 2023-05-28

**Authors:** Cheng Cheng, Peiwen Wang, Yi Yang, Xuan Du, Haibo Xia, Jinyuan Liu, Lu Lu, Hao Wu, Qizhan Liu

**Affiliations:** ^1^ Center for Global Health The Key Laboratory of Modern Toxicology Ministry of Education School of Public Health Suzhou Institute of Public Health Gusu School Nanjing Medical University Nanjing Jiangsu 211166 P. R. China; ^2^ Jiangsu Key Lab of Cancer Biomarkers Prevention and Treatment Collaborative Innovation Center for Cancer Medicine School of Public Health Nanjing Medical University Nanjing Jiangsu 211166 P. R. China; ^3^ Department of Thoracic and Cardiovascular Surgery Jiangsu Province Hospital The First Affiliated Hospital of Nanjing Medical University Nanjing Jiangsu 210029 P. R. China; ^4^ Department of Emergency Jiangsu Province Hospital The First Affiliated Hospital of Nanjing Medical University Nanjing Jiangsu 210029 P. R. China

**Keywords:** circRNAs, N6‐methyladenosine, non‐small cell lung cancer, smoking, tumor‐associated macrophages

## Abstract

Lung cancer is a commonly diagnosed disease worldwide, with non‐small cell lung cancers (NSCLCs) accounting for ≈ 85% of cases. Cigarette smoke is an environmental exposure promoting progression of NSCLC, but its role is poorly understood. This study reports that smoking‐induced accumulation of M2‐type tumor‐associated macrophages (M2‐TAMs) surrounding NSCLC tissues promotes malignancy. Specifically, extracellular vesicles (EVs) from cigarette smoke extract (CSE)‐induced M2 macrophages promoted malignancy of NSCLC cells in vitro and in vivo. circEML4 in EVs from CSE‐induced M2 macrophages is transported to NSCLC cells, where it reduced the distribution of ALKBH5 in the nucleus by interacting with Human AlkB homolog H5 (ALKBH5), resulting in elevated N6‐methyladenosine (m6A) modifications. m6A‐seq and RNA‐seq revealed suppressor of cytokine signaling 2 (SOCS2)‐mediated activation of the Janus kinase‐signal transducer and activator of transcription (JAK‐STAT) pathway by regulating m6A modification of SOCS2 via ALKBH5. Down‐regulation of circEML4 in EVs from CSE‐induced M2 macrophages reversed EVs‐enhanced tumorigenicity and metastasis in NSCLC cells. Furthermore, this study found that smoking patients showed an increase in circEML4‐positive M2‐TAMs. These results indicate that smoking‐induced M2‐TAMs via circEML4 in EVs promote the NSCLC progression through ALKBH5‐regulated m6A modification of SOCS2. This study also reveals that circEML4 in EVs from TAMs acts as a diagnostic biomarker for NSCLC, especially for patients with smoking history.

## Graphical abstract

Smoking promotes the progression of non‐small cell lung cancers (NSCLC) through increasing accumulation of M2‐type tumor‐associated macrophages. Specifically, circEML4 in extracellular vesicles (EVs) from smoking‐induced M2 macrophages regulates the JAK‐STAT signaling axis via ALKBH5‐mediated m6A modification of SOCS2 in NSCLC cells. The results reveal that circEML4 in EVs is a potential biomarker of NSCLC, especially for patients with smoking history.

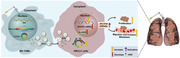

## 1 Introduction

Lung cancer is a highly prevalent cancer worldwide, representing 11.6% of all diagnosis. Deaths related to lung cancer account for 22% of overall cancer mortality.^[^ ^1^ ^]^ Globally, the five‐year relative survival rate for lung cancer is < 21% in both men and women.^[^ ^2^ ^]^ Lung cancer is primarily a disease related to smoking, with smokers having a 10–20% morbidity rate, while never‐smokers have only a 1–2% rate.^[^ ^3^ ^]^ Non‐small cell lung cancers (NSCLCs) account for ≈ 85% of cases, with smokers being at a higher risk of developing NSCLC than never‐smokers, as evidenced by past research.^[^ ^4^ ^]^ Relative to non‐smokers, continuing smokers have a 2.94 times higher risk of all‐cause mortality from NSCLC.^[^ ^5^ ^]^ In addition, the disease‐free survival (DFS) and overall survival (OS) of NSCLC patients with a smoking period ≥ 40 years were shorter than those of patients with a smoking period of < 40 years.^[^ ^6^ ^]^ Epidemiological analysis shows that cigarette smoking is a risk factor for NSCLC development. Nevertheless, the biological mechanisms by which smoking promotes NSCLC progression remain unclear.

Tumor‐associated macrophages (TAMs) are macrophages that infiltrate tumor tissues. In tumor tissues, they are stromal cells, and in the tumor microenvironment, they are the most abundant immune population, accounting for ≈ 50% of hematopoietic cells.^[^ ^7^, ^8^ ^]^ Most human tumor tissues, exhibit elevated infiltration of TAMs, indicating their involvement in the development of tumors.^[^ ^7^ ^]^ Macrophages are classified into the M1 and M2 types. M1 macrophages are components of anti‐tumor immunity, and M2 macrophages are involved in promoting tumor development. TAMs mainly have the characteristics of M2 macrophages.^[^ ^9^, ^10^ ^]^ At primary and metastatic sites, M2‐TAMs drive tumor progression through their effects on basement membrane breakdown and deposition, angiogenesis, leukocyte recruitment, and global immunosuppression.^[^ ^11^ ^]^ TAMs also have biological effects and are potential targets for cancer diagnosis and treatment.^[^ ^12^ ^]^ Although the role of TAMs in cancer has been explored, further clarification is needed on how environmental factors, such as cigarette smoke (CS) exposure, influence NSCLC progression through TAMs.

Intercellular communication mediated by extracellular vesicles (EVs) has been extensively studied in tumors.^[^ ^13^ ^]^ EVs carry non‐coding RNAs (ncRNAs) and have regulatory roles in tumor metastasis, proliferation, and drug resistance.^[^ ^14^ ^]^ EVs, secreted from various cells, including immune and cancer cells, are present in body fluids such as peripheral blood and urine.^[^ ^15^ ^]^ Cell proliferation and invasion are features of malignant tumor progression. TAMs induce tumor proliferation and invasion by the interaction between miR‐95 in EVs and JunB of prostate cancer cells.^[^ ^16^ ^]^ Moreover, nicotine, a component of CS, inhibits innate immune function and promotes brain metastasis from lung cancer.^[^ ^17^ ^]^ Thus, studying ncRNAs in EVs from TAMs could aid in diagnosing and treating smoking‐promoted NSCLC.

N6‐methyladenosine (m6A)‐modified RNAs, which involve methylation of the N6 position of adenosine, regulate transcriptional levels and plays a role in tumor molecular mechanisms.^[^ ^18^ ^]^ Human AlkB homolog H5 (ALKBH5), an m6A‐modified demethylase (eraser), has a regulatory role in various tumors, including lung cancer, gastric cancer, ovarian cancer, and colorectal cancer, by regulating the level of m6A modifications of mRNA.^[^ ^19^, ^20^ ^]^ Since m6A methylation and demethylation occur mainly in the nucleus, examining the nucleocytoplasmic distribution of the methyltransferase is crucial for understanding the role of m6A modification in tumorigenesis.^[^ ^21^ ^]^ Also, the m6A modification is involved in regulating the pro‐tumor and anti‐tumor functions of immune cells in the immune microenvironment,^[^ ^22^ ^]^ and reducing the overall m6A levels affects macrophage reprogramming and promoting growth and metastasis of cancer.^[^ ^23^ ^]^ However, the modification of m6A in NSCLC caused by smoking is unknown.

Activation of the Janus kinase/signal transducer and activator of transcription (JAK/STAT) pathway is a regulatory factor for invasion and migration of liver cancer,^[^ ^24^ ^]^ hematological malignancies,^[^ ^25^ ^]^ breast cancer, and head and neck cancer.^[^ ^26^ ^]^ The suppressor of cytokine signaling (SOCS) protein family is believed to negatively regulate JAK/STAT signaling in multiple diseases.^[^ ^27^ ^]^ Nevertheless, the function of the SOCS protein family in regulating the JAK‐STAT pathway in smoking‐induced NSCLC progression is unclear.

Here, we report the mechanism by which smoking‐induced secretion of EVs by M2‐TAMs promotes the malignant progression of NSCLC. Specifically, the treatment of macrophages with cigarette smoke extract (CSE) caused M2 polarization and elevated circEML4 expression. The EVs released by CSE‐induced M2 macrophages contained circEML4 and were transferred into NSCLC cells, and circEML4 in EVs increased distribution of the ALKBH5 in the cytoplasm by interacting with it. In NSCLC cells, reduced distribution of ALKBH5 in the nucleus results in increased m6A modification of SOCS2 and reduced expression of SOCS2, which stimulates the JAK‐STAT pathway and promotes the NSCLC progression. Our findings indicate that exposure to CS increases the expression of circEML4 in EVs secreted by M2‐TAMs promotes the progression of NSCLC via a m6A modification of SOCS2 mediated by ALKBH5 and stimulates the JAK‐STAT signaling axis.

## 2 Results

### 2.1 Cigarette Smoking is Associated with the Progression of NSCLC and the Accumulation of M2‐TAMs in NSCLC Tissues

**Figure 1:**
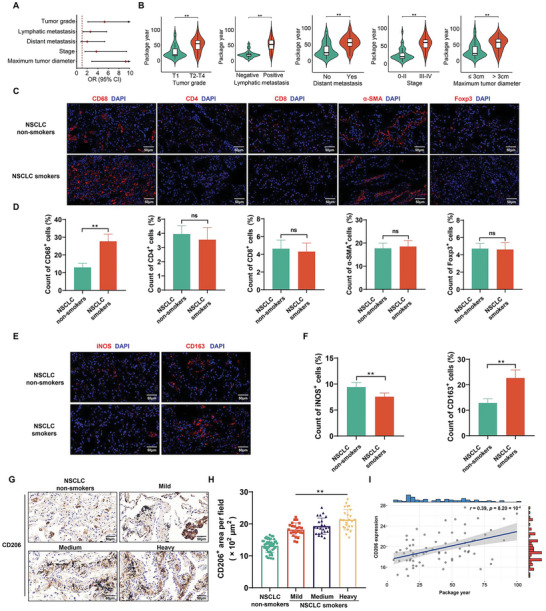
Cigarette smoking is associated with the progression of NSCLC and the accumulation of M2‐TAMs in NSCLC tissues. A) Logistic regression models of associations of cigarette smoking with tumor grade, lymphatic metastasis, distant metastasis, stage, and maximum tumor diameter for individuals with NSCLC. The logistic regression models were adjusted for age and sex (Non‐smokers, *n* = 48; Smokers, *n* = 70). B) Levels of cigarette smoking for individuals with NSCLC with different tumor grades, lymphatic metastasis, distant metastasis, stage, and maximum tumor diameter (*n* = 70). C) Immunofluorescence staining for macrophages (CD68^+^), CD4^+^ T cells, CD8^+^ T cells, CAFs (*α*‐SMA^+^), and Tregs (Foxp3^+^) was measured to reveal different cell types (red) in the tumor microenvironment (*n* = 8), and D) measurement of CD68, CD4, CD8, *α*‐SMA, and Foxp3‐positive cells in NSCLC tissues from smoking or never‐smoking patients (*n* = 8, randomly selected fields per group). DAPI labels nuclei (blue). Scale bar, 50 µm. E) Immunofluorescence staining for iNOS (red) and CD163 (red) was measured (*n* = 8), and F) measurement of iNOS‐and CD163‐positive cells in NSCLC tissues from smoking or never‐smoking patients (*n* = 8, randomly selected fields per group). DAPI labels nuclei (blue). Scale bar, 50 µm. NSCLC patients with a smoking history were divided into groups according to levels of cigarette smoking (Non‐smokers, *n* = 48; Mild group, *n* = 23; Medium group, *n* = 23; Heavy group, *n* = 24). G) IHC staining for CD206 was conducted, and H) measurement of CD206‐positive signals in NSCLC tissues from different levels of cigarette smoking. Scale bar, 50 µm. I) Correlation of CD206 expression in NSCLC tissues for NSCLC patients with different levels of cigarette smoking. All data represent means ± SD. ns, not significant. *p < 0.05; ^**^ *p* < 0.01.

As determined by logistic regression, for NSCLC patients, cigarette smoking was positively associated with tumor grade (OR = 5.28, *p* < 0.01), lymphatic metastasis (OR = 2.59, *p* = 0.02), stage (OR = 3.72, *p* < 0.01), and maximum tumor diameter (OR = 9.27, *p* < 0.01) (**Figure** **1**A). Information about the study population is included in Table S1 (Supporting Information). Higher levels of cigarette smoking were notable in NSCLC patients with T2–T4 tumor grade, positive lymphatic metastasis, distant metastasis, stage III–IV, and a maximum tumor diameter > 3 cm (Figure 1B). Furthermore, we analyzed the relationship between smoking and non‐smoking in patients with NSCLC and a variety of cells in tumor microenvironment. Compared with NSCLC tissues of non‐smokers, CD68^+^ macrophages were up‐regulated in tissues of smoking NSCLC patients. However, there were no significant differences in other cells in tumor microenvironment between smokers and never‐smokers with NSCLC (Figure 1C,D). Also, as shown through evaluation of The Cancer Immunome Atlas, samples of lung adenocarcinoma and lung squamous cell carcinoma showed macrophage enrichment (Figure S1, Supporting Information). These results indicate that macrophages have an essential role in NSCLC. Biomarkers of M1‐TAMs in tumor tissues were tested by iNOS, and M2‐TAMs were tested by CD163 or CD206.^[^ ^28^, ^29^ ^]^ In contrast to non‐smoking NSCLC patients, there was lower expression of iNOS and enhanced expression of CD163 for smoking patients (Figure 1E,F). Moreover, NSCLC smokers demonstrated elevated expression of CD206 compared with NSCLC non‐smokers. In NSCLC tissues, CD206 expression was found to be positively correlated with the levels of cigarette smoking (Figure 1G,H), which was further confirmed by spearman correlations (*r* = 0.39, *p* = 8.20 × 10^−4^) (Figure 1I). Overall, these results reveal that smoking promotes the progression of NSCLC by increasing the accumulation of M2‐TAMs.

### 2.2 CSE Promotes the Progression of NSCLC Cells Through Promoting M2 Polarization of Macrophages in Vitro and in Vivo

**Figure 2:**
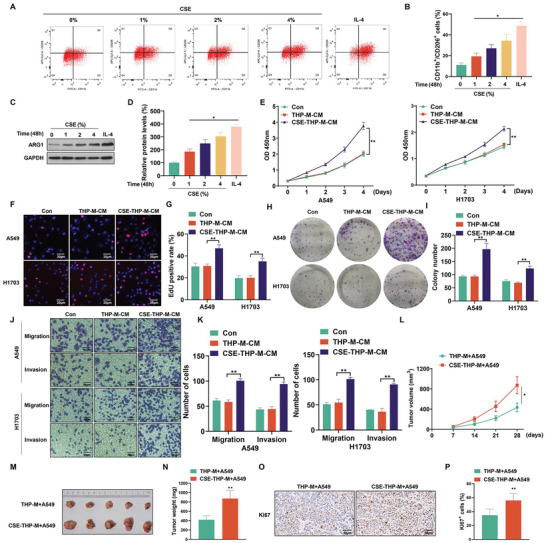
CSE promotes the progression of NSCLC cells through promoting M2 polarization of macrophages in vitro and in vivo. THP‐M were exposed to 0, 1, 2, or 4% CSE for 48 h; IL‐4 was used as a positive control. A) M2 polarization with representative displays and B) the percentage of M2 macrophages as determined by flow cytometry analysis. C) Western blots were performed, and D) the relative protein levels of ARG1 were measured in THP‐M. A549 and H1703 cells were exposed to THP‐M‐CM or CSE‐THP‐M‐CM for 24 h. THP‐M were exposed to 4% CSE for 48 h. The proliferation of A549 and H1703 cells was measured using E) CCK‐8 assays. F) EdU assays and G) EdU‐positive rates were determined. H) Colony formation assays were performed, and I) colony formation by A549 and H1703 cells was assessed. J) Representative images of Transwell assays, and K) migration and invasion were determined for A549 and H1703 cells. A549 cells were mixed with THP‐M or CSE‐THP‐M (5 × 10^6^) and injected into NOD/SCID mice. L) Tumor volume summary for mice, measured every seven days. M) Images of xenograft tumors harvested from NOD/SCID mice. N) NOD/SCID mice (*n* = 5) were examined for tumor weights. O) IHC staining for Ki67 was measured (*n* = 5), and P) measurement of Ki67‐positive signals in tumor tissues harvested from NOD/SCID mice (*n* = 5, randomly selected fields per group). DAPI labels nuclei (blue). Scale bar, 50 µm. Three independent experiments were conducted. All data represent means ± SD. *p < 0.05; ^**^ *p* < 0.01.

There was a higher number of M2 macrophages in CSE‐treated THP‐macrophages (THP‐M) (**Figure** **2**A,B), concomitant with elevated protein expression of ARG1 (Figure 2C,D). Electron microscopy revealed the elongated morphology of THP‐M treated with CSE or interleukin‐4 (IL‐4) (Figure S2A, Supporting Information). In addition, the CSE‐induced M2 polarization of THP‐M was verified by immunofluorescence and qRT‐PCR (Figure S2B,C, Supporting Information). When A549 and H1703 cells were exposed to culture medium (CM) from CSE‐THP‐M (Figure S3, Supporting Information), the proliferation of A549 and H1703 cells was enhanced (Figure 2E). 5‐Ethynyl‐2′‐deoxyuridine (EdU) staining showed that the CM from CSE‐THP‐M increased the numbers of proliferating A549 and H1703 cells (Figure 2F,G). Treatment of A549 and H1703 cells with CM from CSE‐THP‐M resulted in an increase in their colony‐forming capacity (Figure 2H,I). Moreover, compared to the CM from THP‐M, A549 and H1703 cells treated with CM from CSE‐THP‐M had accelerated migration and invasion (Figure 2J,K).

To further determine the role of the CSE‐induced M2 polarization of THP‐M on the tumorigenicity of A549 cells, THP‐M or CSE‐THP‐M were mixed with A549 cells and injected into NOD/SCID mice (Figure S4A, Supporting Information). The results showed that tumors of mice injected with CSE‐THP‐M mixed with A549 cells were larger in volume than those injected with THP‐M mixed with A549 cells (Figure 2L); the weights of tumors were also greater (Figure 2M,N). Additionally, compared with those injected with THP‐M and A549 cells combined, the numbers of Ki67‐positive cells were elevated in tumors of the group injected with CSE‐THP‐M and A549 combined (Figure 2O,P). These results show that CSE‐induced M2 polarization of macrophages and promotes the malignancy of NSCLC cells in vitro and in vivo.

### 2.3 CSE Promotes the Progression of NSCLC Cells Through Secreting EVs from M2 Macrophages in Vitro and in Vivo

**Figure 3:**
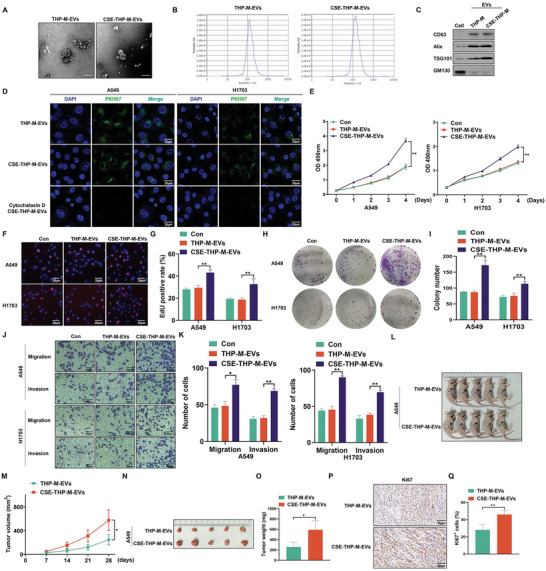
CSE promotes the progression of NSCLC cells through secreting EVs from M2 macrophages in vitro and in vivo. THP‐M were exposed to 4% CSE for 48 h. A) The shape of EVs was characterized by TEM of purified THP‐M‐EVs and CSE‐THP‐M‐EVs. Scale bar, 200 nm. B) The particle number and size analysis of EVs were assessed for THP‐M and CSE‐THP‐M. C) Western blots were performed for cells and EVs. D) Representative images of incubation with THP‐M‐EVs and CSE‐THP‐M‐EVs labeled with PKH67 (green) with A549 and H1703 cells. The proliferation of A549 and H1703 cells was determined by E) CCK‐8 assays. F) EdU assays and G) EdU‐positive rates were determined. H) Colony formation assays were performed, and I) the numbers of colonies were determined for A549 and H1703 cells. J) Representative images of Transwell assays, and K) migration and invasion were determined for A549 and H1703 cells. L) Tumors in the subcutaneous layer were observed in both groups. (The arrows point to tumor xenografts.) M) Tumor volume summary for mice, measured every seven days. N) Images of xenograft tumors harvested from nude mice. O) Nude mice were examined for tumor weights (*n* = 5). P) IHC staining for Ki67 was measured (*n* = 5), and Q) measurement of Ki67‐positive signals in tumor tissues harvested from nude mice (*n* = 5, randomly selected fields per group). Scale bar, 50 µm. Three independent experiments were conducted. All data represent means ± SD. *p < 0.05; ^**^ *p* < 0.01.

Analysis of EVs revealed nanovesicles with diameters of ≤ 200 nm, and the particle sizes and count distributions were similar between THP‐M‐EVs and CSE‐THP‐M‐EVs (**Figure** **3**A,B). Furthermore, in EVs, there was high expression of CD63, Alix, and TSG101 and minimal expression of GM130 (Figure 3C). These results verified the characteristics of the EVs. The pattern of THP‐M‐derived EVs for treating NSCLC cells was shown in Figure S3 (Supporting Information). Furthermore, A549 and H1703 cells were observed to absorb EVs labeled with PKH67 from both THP‐M and CSE‐THP‐M. However, in the presence of cytochalasin D, which inhibits uptake of EVs, this absorption was eliminated (Figure 3D). EVs from CSE‐THP‐M increased the proliferation of A549 and H1703 cells (Figure 3E). EdU assay also proved that CSE‐THP‐M‐derived EVs promoted the proliferation of A549 and H1703 cells (Figure 3F,G). Furthermore, the colony‐forming (Figure 3H,I), migration, and invasion (Figure 3J,K) capacities of A549 and H1703 cells were enhanced by treatment with EVs from CSE‐THP‐M.

To explore the roles of EVs from CSE‐THP‐M in vivo, tumorigenicity assays were conducted in nude mice (Figure S4B, Supporting Information). EVs from CSE‐THP‐M caused increases in volumes (Figure 3L,M) and weights (Figure 3N,O) of tumors produced by subcutaneous implantation of A549 cells. In addition, compared with treatment of the THP‐M‐EVs group, the numbers of Ki67‐positive cells in tumors of the CSE‐THP‐M‐EVs treated group were elevated (Figure 3P,Q). Furthermore, compared to those without the injection of EVs from CSE‐THP‐M, the volumes (Figure S5A,B, Supporting Information), weights (Figure S5C,D, Supporting Information), and numbers of Ki67‐positive cells (Figure S5E,F, Supporting Information) were elevated in tumors of the group injected with EVs from CSE‐THP‐M. Inhibition of EVs secretion from CSE‐THP‐M with GW4869, which blocks EVs production, reversed the CM‐promoted proliferation (Figure S6A–C, Supporting Information), colony formation (Figure S6D,E, Supporting Information), migration, and invasion (Figure S6F,G, Supporting Information) of A549 and H1703 cells. Collectively, these findings indicate that EVs are essential for CSE‐induced M2 macrophages to promote progression of NSCLC cells.

### 2.4 circEML4 in EVs from CSE‐induced M2 Macrophages Promotes the Proliferation, Migration, and Invasion of NSCLC Cells

**Figure 4:**
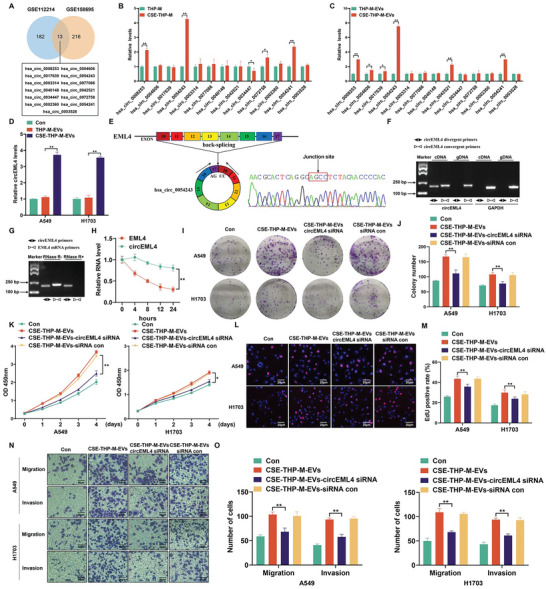
circEML4 in EVs from CSE‐induced M2 macrophages promotes the proliferation, migration, and invasion of NSCLC cells. A) Venn diagram exhibiting the 13 common circRNAs between the GSE112214 and GSE158695. circRNAs with significant upregulation were defined as *p* < 0.05 and FC > 1.5. B) qRT‐PCR to determine expression of 13 common circRNAs in THP‐M and CSE‐THP‐M. THP‐M were exposed to 4% CSE for 48 h. C) qRT‐PCR to determine levels of the 13 common circRNAs in EVs from THP‐M and CSE‐THP‐M. D) circEML4 levels were determined by qRT‐PCR in A549 and H1703 cells after 24 h treatment with THP‐M‐EVs or CSE‐THP‐M‐EVs. E) The formation of circEML4 is illustrated in the schematic. The circEML4 back‐splicing junction was identified through Sanger sequencing. F) circEML4 was present in THP‐M, as determined by RT‐PCR using convergent and divergent primers. GAPDH served as a negative control. G) RT‐PCR confirmation of circEML4 stability after RNase R treatment. H) Upon treatment with actinomycin D, the half‐lives of circEML4 and linear EML4 were measured. I) Colony formation assays were performed, and J) colony formation was assessed for A549 and H1703 cells. The proliferation of A549 and H1703 cells was measured by K) CCK‐8 assays. L) EdU assays and M) EdU‐positive rates were determined. N) Representative images of Transwell assays, and O) migration and invasion were determined for A549 and H1703 cells. All data represent means ± SD. *p < 0.05; ^**^ *p* < 0.01.

To assess the effect of circRNAs on the development of NSCLC, 13 circRNAs over‐expressed in NSCLC tissues were acquired from the GSE112214 and GSE158695 databases (**Figure** **4**A and Table S2, Supporting Information). We then measured the expression of circEML4 (hsa_circ_0054243) in the EVs secreted by THP‐M and CSE‐THP‐M and found that the expression of circEML4 was upregulated in CSE‐THP‐M and in EVs secreted by CSE‐THP‐M (Figure 4B,C). In addition, the levels of circEML4 were up‐regulated in A549 and H1703 cells treated with EVs from CSE‐THP‐M (Figure 4D). As reported in Circbank, circEML4 was derived from exon 10 and exon 17 of the EML4 gene. Sanger sequencing further confirmed the existence of spliced junctions of circEML4 (Figure 4E). Convergent and divergent primers were designed to amplify EML4 mRNA and circEML4 in cDNA and gDNA from THP‐M (Figure 4F). The results demonstrated that circEML4 could be detected in cDNA, but not in gDNA. In addition, circEML4 was resistant to RNase R treatment, and linear EML4 mRNA was diminished (Figure 4G). After treatment with actinomycin D, the half‐life of circEML4 was found to be longer than that of linear EML4 (Figure 4H). These results show that cirEML4 is stable and is not readily degraded by RNases.

Next, the circEML4 siRNA was used to knockdown circEML4 in EVs from CSE‐THP‐M. A circEML4 siRNA with the highest knockdown efficiency was chosen (Figure S7, Supporting Information). The results showed that EVs from CSE‐THP‐M cells transfected with circEML4 siRNA led to a reduction in the colony‐forming capacity of A549 and H1703 cells (Figure 4I,J). CCK8 and EdU assays showed that down‐regulation of circEML4 in EVs from CSE‐THP‐M reversed the EVs‐increased proliferation of A549 and H1703 cells (Figure 4K–M). Lastly, A549 and H1703 cells treated with EVs from CSE‐THP‐M were transfected with circEML4 siRNA, which resulted in reduced cell migration and invasion of A549 and H1703 cells (Figure 4N,O). Thus, these results confirm that CSE‐induced M2 macrophages promote the malignant progression of NSCLC cells through circEML4 in EVs.

### 2.5 circEML4 in EVs from CSE‐induced M2 Macrophages Regulates the Distribution of ALKBH5 through Combining with ALKBH5 and Increases the Levels of m6A in NSCLC Cells

**Figure 5:**
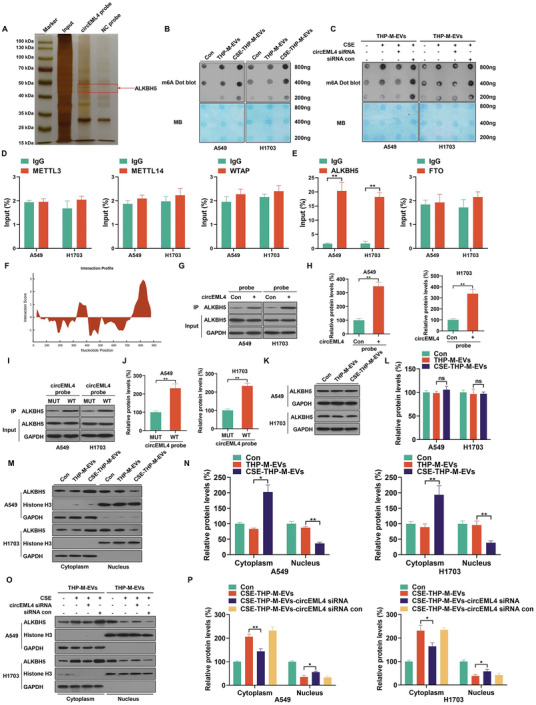
circEML4 in EVs from CSE‐induced M2 macrophages regulates the distribution of ALKBH5 through combining with ALKBH5 and increasing the levels of m6A in NSCLC cells. A) Silver staining of circEML4 pulldown. THP‐M were exposed to 4% CSE for 48 h. B,C) RNA m6A dot blot assays were used to assess the m6A levels of total mRNA. Methylene blue staining was used to determine the loading control. The interactions of circEML4 with the m6A writer D) proteins (METTL3, METTL14, and WTAP) and eraser E) proteins (ALKBH5 and FTO) were determined by RIP assays of A549 and H1703 cells. F) Interaction propensity prediction between circEML4 and ALKBH5 by CatRAPID. G,H) the circEML4 and ALKBH5 interaction was verified by RNA pull‐down assays with A549 and H1703 cells. circEML4 plasmid (2 µg) was transfected into 5 × 10^5^ cells using Lipofectamine 2000. I,J) a circEML4 Mut plasmid or a WT plasmid was transfected into A549 and H1703 cells, and then a circEML4 probe was used for RNA‐pulldown assays. K) Western blots were performed, and L) relative protein expression of ALKBH5 was measured for A549 and H1703 cells. M) Western blots were performed, and N) nuclear and cytoplasmic localizations of ALKBH5 were quantified for A549 and H1703 cells. O) Western blots were performed, and P) nuclear and cytoplasmic localizations of ALKBH5 were quantified for A549 and H1703 cells. Three independent experiments were conducted. All data represent means ± SD. *p < 0.05; ^**^ *p* < 0.01.

RNA immunoprecipitation (RIP) assays with the AGO2 antibody showed no significant difference between the AGO2 and IgG groups in A549 and H1703 cells, suggesting that circEML4 was not a miRNA sponge (Figure S8, Supporting Information). Using RNA pull‐down and mass spectrometry assays to identify circEML4‐interacting proteins, we found that circEML4 interacted with ALKBH5 (**Figure** **5**A and Table S3, Supporting Information). Besides, m6A dot blot assays established that CM from CSE‐THP‐M increased the m6A levels of A549 and H1703 cells (Figure S9A, Supporting Information). Similar results were observed in A549 and H1703 cells treated with EVs isolated from CSE‐THP‐M (Figure 5B). m6A dot blot assays also demonstrated that inhibition of the circEML4 or EVs secretion by CSE‐THP‐M reversed EVs‐ or CM‐increased m6A levels in A549 and H1703 cells (Figure 5C; Figure S9B, Supporting Information). Therefore, ALKBH5 was selected for further experiments. Furthermore, fluorescence in situ hybridization (FISH) experiments confirmed that the uptake of circEML4 by A549 and H1703 cells increased after treatment with EVs from CSE‐THP‐M (Figure S10, Supporting Information). Using RIP assays, we found that circEML4 binds to ALKBH5 in A549 and H1703 cells (Figure 5D,E). Using CatRAPID, the binding sites for circEML4 and ALKBH5 were predicted (Figure 5F); the specific site information is shown in Figure S11A–C (Supporting Information). RNA pull‐down assays showed that more ALKBH5 was pulled down by a circEML4 probe than by a control probe (Figure 5G,H). Further, binding was enhanced in the circEML4 wild‐type group in A549 and H1703 cells as compared to that in the circEML4 mutation group (Figure 5I,J). These data indicate that CSE‐induced M2 macrophages enhance the m6A levels in NSCLC cells through circEML4 binding to ALKBH5.

However, there was no appreciable effect on ALKBH5 protein expression in A549 or H1703 cells treated with EVs from THP‐M or CSE‐THP‐M (Figure 5K,L). Elevated binding of circEML4 and ALKBH5 was evident in A549 and H1703 cells treated with EVs from CSE‐THP‐M (Figure S12A, Supporting Information). When A549 and H1703 cells were exposed to EVs from CSE‐THP‐M, the distribution of ALKBH5 was enhanced in the cytoplasm and reduced in the nucleus (Figure 5M,N). Additionally, the binding amounts of circEML4 and ALKBH5 were reduced in A549 and H1703 cells after treatment with EVs from CSE‐THP‐M and transfection with circEML4 siRNA (Figure S12B, Supporting Information); reduced distribution of ALKBH5 in the cytoplasm was also evident (Figure 5O,P). These results support the effect of circEML4 in EVs from CSE‐induced M2 macrophages in enhancing the distribution of ALKBH5 in the cytoplasm of NSCLC cells by combining with ALKBH5 and elevating the levels of m6A.

### 2.6 circEML4 in EVs from CSE‐induced M2 Macrophages Regulates m6A Modification of SOCS2 via ALKBH5 in Promoting the Progression of NSCLC Cells

**Figure 6:**
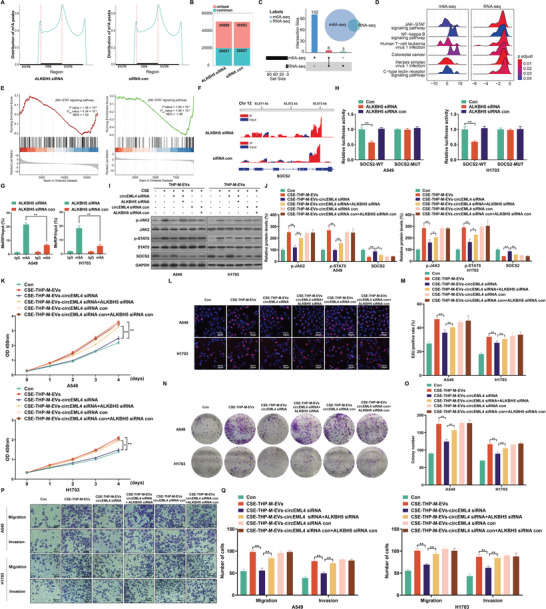
circEML4 in EVs from CSE‐induced M2 macrophages regulates m6A modification of SOCS2 via ALKBH5 in promoting the progression of NSCLC cells. A) The distribution of m6A peaks over the length of mRNA transcripts was analyzed for A549 cells transfected with ALKBH5 siRNA or siRNA con. B) Numbers of m6A peaks revealed by m6A‐seq from A549 cells transfection with ALKBH5 siRNA or siRNA con. C) The number of pathways by GSEA enrichment analysis at m6A and mRNA levels. D) The distribution of the core genes of the enrichment pathway was displayed, and each gene's log2 fold change was represented on the x‐axis. > 0 represented up‐regulated expression; < 0 represented down‐regulated expression. E) GSEA enrichment plot of the JAK‐STAT signaling pathway recognized by m6A‐seq and RNA‐Seq. F) Peaks demonstrating the abundance of m6A sites in SOCS2 mRNA. G) MeRIP‐qPCR determinations for m6A enrichment on SOCS2 mRNA in A549 and H1703 cells. H) Relative activities of the WT and Mut luciferase reporters. THP‐M were exposed to 4% CSE for 48 h. I) Western blots were performed, and J) relative protein levels of p‐JAK2, p‐STAT5, and SOCS2 were measured for A549 and H1703 cells. The proliferation of A549 and H1703 cells was assessed by K) CCK‐8 assays. L) EdU assays and M) EdU‐positive rates were determined. N) Colony formation assays were performed, and O) colony formation was determined for A549 and H1703 cells. P) Representative images of Transwell assays, and Q) migration and invasion were determined for A549 and H1703 cells. Three independent experiments were conducted. All data represent means ± SD. *p < 0.05; ^**^ *p* < 0.01.

To determine ALKBH5‐mediated mechanisms, we conducted m6A‐seq and RNA‐seq assays on A549 cells transfected with ALKBH5 siRNA or siRNA control. In these cells, m6A peaks were enriched in the 3’ untranslated regions (UTRs) (**Figure** **6**A; Figure S13A, Supporting Information). These cells displayed an enriched consensus motif within m6A (Figure S13B, Supporting Information). In A549 cells, 30880 new m6A peaks appeared after the ALKBH5 knockdown (Figure 6B). By GSEA enrichment analysis of genes differentially expressed at m6A levels and mRNA levels, six common enrichment pathways were identified (Figure 6C and Tables S4 and S5, Supporting Information). The JAK‐STAT signaling pathway was selected for further study owing to its high score (Figure 6D,E). Next, the expressions of genes enriched in the JAK‐STAT pathway were analyzed. In A549 cells, the mRNA levels of SOCS2 were reduced by ALKBH5 knockdown according to mRNA‐seq results (Figure S13C, Supporting Information). A pan‐cancer analysis revealed that SOCS2 levels were down‐regulated in numerous cancers (Figure S14A, Supporting Information). For lung cancer, a KM Plotter tool showed a correlation between low SOCS2 expression and poor overall survival (Figure S14B, Supporting Information). Further, analysis of six GSE datasets revealed that the expression of SOCS2 was lower from smokers than in those from non‐smokers (Figure S14C, Supporting Information). Therefore, we reasoned that SOCS2 was a target gene.

The m6A modification site of SOCS2 in 5’ UTRs was obtained from the m6A‐seq data (Figure 6F). In addition, meRIP‐qPCR assays showed that, in A549 and H1703 cells, the m6A levels of SOCS2 were up‐regulated after transfection with ALKBH5 siRNA (Figure 6G). The m6A levels of SOCS2 in A549 and H1703 cells were also enhanced by treatment with EVs from CSE‐THP‐M (Figure S15, Supporting Information). This indicates that, in NSCLC cells, EVs from CSE‐THP‐M participate in regulating the m6A levels of SOCS2. To identify the m6A modification site on SOCS2, we mutated the m6A site of the SOCS2 5’ UTR to construct a mutant SOCS2 (Figure S16, Supporting Information). After transfection with ALKBH5 siRNA, the WT group had lower luciferase activity, but not the mutant group (Figure 6H). For A549 and H1703 cells, knockdown of ALKBH5 reduced levels of SOCS2 at the protein level (Figure S17A,B, Supporting Information). These findings indicate that ALKBH5 regulates SOCS2 expression through m6A modifications.

SOCS2 regulates JAK‐STAT signaling in cancer cells.^[^ ^30^, ^31^ ^]^ Our data indicated that low expression of circEML4 attenuated the upregulation of p‐JAK2 and p‐STAT5 and the downregulation of SOCS2 induced by EVs from CSE‐THP‐M (Figure S18A,B, Supporting Information). Additionally, in A549 and H1703 cells, an ALKBH5 siRNA reversed the effects of EVs from CSE‐THP‐M transfected with circEML4 siRNA on the expression of p‐JAK2, p‐STAT5, and SOCS2 (Figure 6I,J). The CCK8 and EdU assays further showed that knockdown of circEML4 in EVs from CSE‐THP‐M cells inhibited the proliferation of A549 and H1703 cells. However, this inhibition was restored by knockdown of ALKBH5 (Figure 6K–M). EVs from CSE‐THP‐M transfected with circEML4 siRNA inhibited clone formation (Figure 6N,O), migration, and invasion (Figure 6P,Q) of A549 and H1703 cells, but these effects were alleviated by an ALKBH5 siRNA. In sum, in NSCLC cells, circEML4 in EVs from CSE‐induced M2 macrophages promotes the progression of NSCLC cells and regulates m6A modification of SOCS2 via ALKBH5.

### 2.7 Inhibition of circEML4 Prevents the Progression of NSCLC Cells Induced by EVs from CSE‐induced M2 Macrophages in Vivo

**Figure 7:**
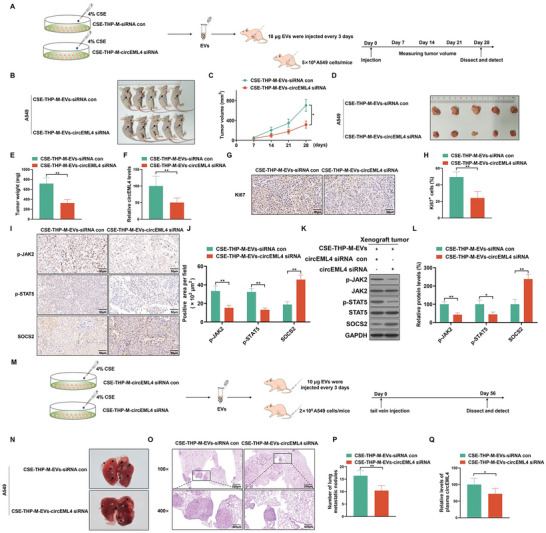
Inhibition of circEML4 prevents the progression of NSCLC cells induced by EVs from CSE‐induced M2 macrophages in vivo. A) Schematic representation of the xenograft nude mice model. B) Tumors of the subcutaneous layer were observed for the two groups. (The arrows point to tumor xenografts.) C) Tumor volume summary for mice, measured every 7 days. D) Images of xenograft tumors harvested from nude mice. E) Nude mice were examined for tumor weights (*n* = 5). F) Levels of circEML4 were assessed by qRT‐PCR in xenograft tumors harvested from nude mice. G) IHC staining for Ki67 was measured (*n* = 5), and H) measurement of Ki67‐positive signals in xenograft tumors harvested from nude mice (*n* = 5, randomly selected fields per group). Scale bar, 50 µm. I) IHC staining for p‐JAK2, p‐STAT5, and SOCS2 was assessed (*n* = 5), and J) measurement of p‐JAK2, p‐STAT5, and SOCS2‐positive signals in xenograft tumors harvested from nude mice (*n* = 5, randomly selected fields per group). Scale bar, 50 µm. K) Western blots were performed, and L) relative protein levels of p‐JAK2, p‐STAT5, and SOCS2 were measured in xenograft tumors harvested from nude mice. M) Schematic illustration of tail vein injection for lung metastasis in a nude mice model. N) Representative lungs were acquired from the nude mice (*n* = 5). O) Representative images of metastatic nodules and P) measurement of metastatic nodules of the lungs from the mice were examined by hematoxylin‐eosin staining (*n* = 5). Q) By qRT‐PCR, levels of circEML4 were assessed in plasma from nude mice (*n* = 5). All data represent means ± SD. *p < 0.05; ^**^ *p* < 0.01.

To investigate the effect of circEML4 in EVs from CSE‐induced M2 macrophages on NSCLC cells, we generated subcutaneous tumors in Balb/c nude mice. The tumors were established using A549 cells with EVs from CSE‐THP‐M or circEML4 downregulated CSE‐THP‐M. A549 cells (5 × 10^6^) were injected into nude mice. EVs (10 µg) from CSE‐THP‐M treated with siRNA control or circEML4 siRNA were injected subcutaneously into nude mice every 3 days to determine the effect of circEML4 on EVs (**Figure** **7**A). EVs from the CSE‐THP‐M‐circEML4 siRNA group caused a decrease in tumor volumes (Figure 7B,C) and weights (Figure 7D,E) of tumors as compared to the control group. Also, compared to the control group, the expression of circEML4 in tumors of nude mice from the CSE‐THP‐M‐circEML4 siRNA group was lower (Figure 7F). Moreover, as determined by immunohistochemical (IHC) staining, mice in this group showed lower levels of Ki67, p‐JAK2, and p‐STAT5 and higher expression of SOCS2 than the control group (Figure 7G–J). The results of protein analyses were consistent with the IHC results (Figure 7K,L).

Furthermore, the role of EVs secreted by CSE‐induced M2 macrophages on NSCLC cells was explored by tail vein injection, as shown in Figure 7M. EVs from circEML4 down‐regulated CSE‐THP‐M were injected into mice; fewer metastatic nodules were formed in the lungs than using EVs from CSE‐THP‐M (Figure 7N), as supported by hematoxylin‐eosin staining (Figure 7O,P). Measurement of the circEML4 expression in plasma of mice demonstrated that circEML4 was reduced in mice treated with EVs from the CSE‐THP‐M‐circEML4 siRNA group compared to the CSE‐THP‐M‐siRNA control group (Figure 7Q). These results confirm that circEML4 in EVs from CSE‐induced M2 macrophages is involved in promoting the growth and metastasis of NSCLC cells.

### 2.8 In NSCLC, Cigarette Smoking is Associated with Activation of the SOCS2/JAK‐STAT Signaling Axis and Increases of circEML4 in M2‐TAMs, Plasma EVs, and NSCLC Tissues

**Figure 8:**
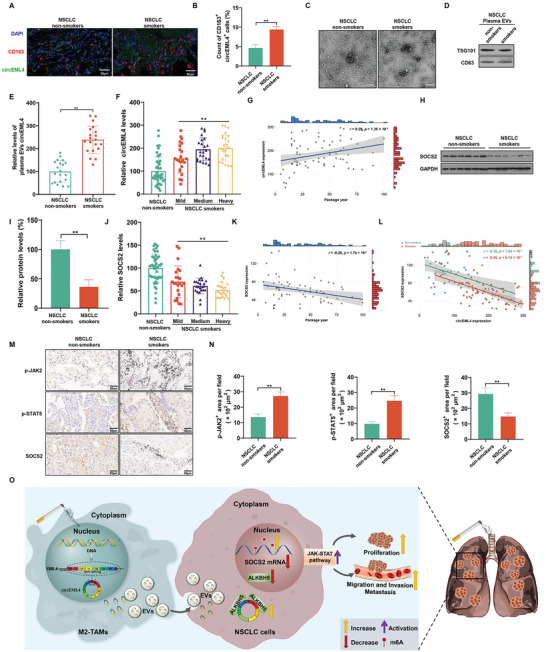
In NSCLC, cigarette smoking is associated with activation of the SOCS2/JAK‐STAT signaling axis and increases of circEML4 in M2‐TAMs, plasma EVs, and NSCLC tissues. A) Immunofluorescence staining for CD163 (red) and circEML4 (green) was measured (*n* = 8), and B) measurement of CD163 and circEML4 double‐positive cells in NSCLC tissues from smoking or never‐smoking patients (*n* = 8, randomly selected fields per group). DAPI labels nuclei (blue). Scale bar, 50 µm. C) The shape of EVs was identified by TEM of purified plasma EVs from smoking or never‐smoking NSCLC patients. Scale bar, 200 nm. D) Western blots were performed for EVs. E) By qRT‐PCR, levels of circEML4 were assessed in NSCLC plasma EVs harvested from smoking or never‐smoking patients (*n* = 20). NSCLC patients with a smoking history were divided into groups according to levels of cigarette smoking (Non‐smokers, *n* = 48; Mild group, *n* = 23; Medium group, *n* = 23; Heavy group, *n* = 24). F) Levels of circEML4 were assessed by qRT‐PCR in NSCLC tissues from smoking or never‐smoking patients. G) Correlation of circEML4 expression in NSCLC tissues with levels of cigarette smoking by NSCLC patients. H) Western blots were conducted, and I) relative protein expression of SOCS2 was measured (*n* = 6). J) By qRT‐PCR, expression of SOCS2 was measured in NSCLC tissues from smoking or never‐smoking patients. K) Correlation of SOCS2 expression in NSCLC tissues with levels of cigarette smoking for NSCLC patients. L) Correlation analyses of expression between circEML4 and SOCS2 in NSCLC tissues from smoking or never‐smoking patients. M) IHC staining for p‐JAK2, p‐STAT5, and SOCS2 was performed (*n* = 8), and N) p‐JAK2, p‐STAT5, and SOCS2 positive signals in NSCLC tissues from smoking or never‐smoking patients were measured (*n* = 8, randomly selected fields per group). Scale bar, 50 µm. O) Schematic illustration of the regulation of smoke‐promoted progression of NSCLC by stimulation of the ALKBH5/SOCS2/JAK‐STAT signaling axis via EVs circEML4 from M2‐TAMs. All data represent mean ± SD. *p < 0.05; ^**^ *p* < 0.01.

FISH assays were used to measure circEML4 expression and CD163 expression to label M2‐TAMs in NSCLC tissues. In contrast to non‐smoking NSCLC patients, there was an increase in expression of circEML4‐positive M2‐TAMs for smoking patients (**Figure** **8**A,B). To assess the levels of circEML4 in plasma EVs from NSCLC patients, plasma EVs were collected from smoking and non‐smoking NSCLC patients. Plasma EVs from patients with NSCLC were characterized by electron microscopy (Figure 8C), particle size analysis (Figure S19, Supporting Information), and protein marker expression analysis (Figure 8D). Compared to the levels of circEML4 in plasma EVs from non‐smoking patients, higher levels were observed in smokers with NSCLC (Figure 8E). Furthermore, in NSCLC tissues of smoking patients, the expression of circEML4 increased with levels of cigarette smoking (Figure 8F,G). Further, SOCS2 protein was lower in the tissues of smoking NSCLC patients compared to never‐smoking patients (Figure 8H,I). For NSCLC tissues, the expression of SOCS2 decreased with levels of cigarette smoking (Figure 8J,K). In addition, circEML4 was negatively associated with SOCS2 in NSCLC tissues of smoking patients (Figure 8L). Finally, when compared with the non‐smoking NSCLC patients, expression of p‐JAK2 and p‐STAT5 were up‐regulated, and expression of SOCS2 was down‐regulated in NSCLC patients who smoked (Figure 8M,N). These findings reveal that circEML4 in EVs from M2‐TAMs is involved in CS promotion of NSCLC through the SOCS2/JAK‐STAT signaling axis.

## 3 Discussion

Globally, smoking is one of the most relevant risk factors for death of NSCLC. In men, 75.5% of lung cancer deaths and, in women, 36.7% of lung cancer deaths are attributed to smoking.^[^ ^32^ ^]^ In addition, smoking is associated with a poor prognosis after surgical procedures for NSCLC.^[^ ^33^ ^]^ In the current study, our results indicated that the TNM stage of patients with NSCLC who smoked was higher than that of non‐smoking NSCLC patients, and the smoking levels of NSCLC patients with high‐grade TNM stage and maximum tumor diameters were higher compared with non‐smoking NSCLC patients.

Immune cells in the tumor microenvironment (TME) mediate the process of tumor metastasis through antitumor and pro‐tumor effects.^[^ ^34^ ^]^ Among these immune cells, cancer‐associated fibroblasts (CAFs) promote the metastasis, invasion, and migration of tumor cells by secreting various cytokines or EVs, and by inhibiting immune function.^[^ ^35^ ^]^ Regulatory T cells (Tregs) are involved in promotion of tumor growth and development, leading to the development of metastatic tumors.^[^ ^36^ ^]^ Macrophages, derived from circulatory monocytes, are an important source of TAMs in cancer microenvironments.^[^ ^37^ ^]^ TAMs promote tumor malignancy and can be categorized into M1 and M2 types. As they play key roles in tumor development, macrophages are considered to be therapeutic targets.^[^ ^38^, ^39^ ^]^ In the TME, TAMs mainly have M2‐like tumor‐promoting effects and participate in various malignant characteristics, including immunosuppression and metastasis.^[^ ^39^, ^40^ ^]^ We analyzed the levels of various immune cells in the tissues of smoking and non‐smoking NSCLC patients. Macrophage expression was higher in NSCLC patients who smoked. Moreover, the numbers of M2‐TAMs in NSCLC tissues were higher for smokers compared with non‐smokers, indicating that smoking‐induced M2 polarization of macrophages has a regulatory role in the development of NSCLC. CS increases the inflammatory response in lung tissues and macrophage recruitment.^[^ ^41^, ^42^ ^]^ The current results suggest that the recruitment of more macrophages around NSCLC tissue by CS may be related to increased levels of inflammation in lung tissue.

TAMs regulate the malignant phenotype of tumor cells through intercellular communication, which is mediated by the ncRNAs of EVs.^[^ ^43^ ^]^ M2 macrophage‐derived EVs carry miR‐21‐5p and miR‐155‐5p and then promote the malignant phenotype of colorectal cancer cells through low expression of BRG1.^[^ ^44^ ^]^ In pancreatic cancer, M2 macrophages secrete miR‐501‐3p‐containing EVs to inhibit TGFBR3 expression and activate the TGF‐*β* signaling pathway, which is associated with the development of pancreatic ductal adenocarcinoma.^[^ ^45^ ^]^ In the present study, CSE induced an increase in M2 polarization of macrophages, with concomitant upregulation of circEML4. Additionally, up‐regulation of circEML4 was evident in the EVs secreted from CSE‐induced M2 macrophages. EVs from CSE‐induced M2 macrophages promoted the malignant phenotype of NSCLC cells. However, the inhibition of EVs or low expression of circEML4 reversed these changes. These findings suggest that circEML4 is transferred from CSE‐induced M2 macrophages to NSCLC cells via EVs, ultimately resulting in an increase in the malignant phenotype of NSCLC cells.

As part of RNA processing, m6A modification participates in export, translation, splicing, and degradation.^[^ ^46^, ^47^ ^]^ In bladder cancer cells, circPTPRA binds to IGF2BP1 to regulate m6A modification and affects the degree of tumor malignancy.^[^ ^48^ ^]^ Our results showed that circEML4 binds to ALKBH5, increasing the distribution of ALKBH5 in the cytoplasm and decreasing its distribution in the nucleus, thereby increasing the m6A modification levels in NSCLC cells. circRNAs regulate m6A modifications that influence downstream target genes to induce tumorigenesis and cancer metastasis. Our findings are consistent with those of previous studies, indicating the regulatory effect of circRNAs on m6A modifications.^[^ ^49^ ^]^ Moreover, m6A‐seq and RNA‐seq identified SOCS2‐mediated activation of the JAK‐STAT signaling pathway by interfering with ALKBH5 expression. Abnormal regulation of the JAK‐STAT pathway participates in the pathogenesis of various malignant tumors.^[^ ^50^, ^51^, ^52^ ^]^ Further, as a negative regulator, SOCS2 is involved in activation of the JAK‐STAT signaling pathway.^[^ ^53^, ^54^ ^]^ For A549 and H1703 cells, down‐regulated circEML4 in EVs changed the over‐expression of p‐JAK2 and p‐STAT5 and the down‐expression of SOCS2 caused by EVs from CSE‐induced M2 macrophages; however, low expression of ALKBH5 alleviated the effect of the circEML4 siRNA. Similar effects were on the proliferation, migration, and invasion of NSCLC cells. Furthermore, the role of M2 macrophage‐derived circEML4 in EVs in regulating the SOCS2/JAK‐STAT signaling axis and promoting malignant progression was validated for a human population.

EVs carry proteins and RNAs into the circulatory system and body fluids. Within EVs, circRNAs have been found to be highly stable and conserved.^[^ ^55^ ^]^ circRNAs in EVs not only retain their functions but also participate in complex intercellular communications within the TME.^[^ ^56^, ^57^ ^]^ Therefore, EVs are considered as biomarkers for diagnosis of various diseases, including tumors.^[^ ^58^, ^59^ ^]^ Engineered EVs can be used as drug therapy vectors, and lncRNA MEG3 in EVs can be used as potential therapeutic target for osteosarcoma.^[^ ^60^ ^]^ Our results suggest that circEML4 in EVs is a target for regulating malignant progression of NSCLC. As shown by our results, reducing the levels of circEML4 in the EVs secreted by CSE‐induced M2 macrophages reduced EV‐enhanced tumorigenicity and the metastatic capacity of NSCLC cells. We found that circEML4 levels were elevated in M2‐TAMs of NSCLC tissues from smokers compared to those from non‐smokers. These results suggest that circEML4 in EVs is a potential diagnostic indicator for NSCLC patients, especially those with smoking history.

In summary, the M2 polarization of macrophages exposed to CS leads to the accumulation of M2‐TAMs surrounding the NSCLC tissues, along with the upregulation of circEML4 expression. circEML4 is transported from M2‐TAMs to NSCLC cells via EVs. In NSCLC cells, reduction of ALKBH5 in the nucleus results in an increase of m6A modification of SOCS2, and SOCS2 levels are down‐regulated, further activating the JAK‐STAT signaling pathway and resulting in increased malignant progression of NSCLC cells (Figure 8O). Our results indicate that, in NSCLC cells, smoking‐induced M2‐TAMs via circEML4 of EVs, promote the progression of NSCLC through ALKBH5‐regulated m6A modification of SOCS2. These results reveal biological insights for circEML4 in EVs derived from TAMs in promoting the progression of NSCLC and providing a diagnostic biomarker for NSCLC, especially among patients with smoking history.

## 4 Experimental Section

### Patients and Specimens

Among the NSCLC tissue samples collected from the First Affiliated Hospital of Nanjing Medical University, 48 were from non‐smokers and 70 were from smokers. The inclusion criteria were as follows: (1) clear pathological diagnosis of NSCLC, (2) neither radiotherapy nor chemotherapy was administered before surgery, (3) no immune system diseases, and (4) with complete data. Tissues were obtained during surgery, fixed by formalin, snap‐frozen in liquid nitrogen, and stored at −80°C. Two pathologists independently confirmed all tumorous tissues. Clinical cancer characteristics were collected from clinical reports. Demographic data, (e.g., smoking history, sex, and age) were acquired by face‐to‐face interviews. This study was approved by the Medical Ethics Committee of First Affiliated Hospital of Nanjing Medical University (no. 2021‐SR‐028).

### Histology, Immunofluorescence, and Immunohistochemistry

Paraffin‐embedded tissues with a thickness of 5 µm were mounted on glass slides and baked at 60°C for 1 h, followed by sequential dehydration in ethanol (Shanghai, China). Before dehydration with ethanol, tissue slides were stained with hematoxylin (Mayer's) and eosin, and mounted with neutral balsam. A light microscope (Leica DM IL LED, Germany) was used to evaluate stained tissues. For paraffin‐embedded tissue sections, immunofluorescence staining was performed using anti‐CD206, anti‐Ki67, anti‐p‐JAK2, anti‐p‐STAT5, and anti‐SOCS2 antibodies (Table S6, Supporting Information). IHC staining of paraffin‐embedded tissue sections was performed with anti‐CD206 antibody, anti‐Ki67 antibody, anti‐p‐JAK2 antibody, anti‐p‐STAT5 antibody, or anti‐SOCS2 antibody (Table S6, Supporting Information). Visualization was performed using HP IHC detection kits (Absin, China). Fluorescence microscopy was used to acquire positive signals. Adobe Photoshop CS was used to investigate the images further.

### Western Blots

The total proteins from tissues or cells were harvested using RIPA Lysis Buffer. Protein concentrations were determined with BCA protein assay kits (Beyotime Biotechnology, China). Samples were separated on 10% SDS‐PAGE gels and transferred to polyvinylidene difluoride membranes (Millipore, USA). Blocking the membranes with 5% nonfat dry milk for 1 h at room temperature was followed by overnight incubations at 4°C with primary antibodies (Table S6, Supporting Information). The following day, an additional incubation period of 1 h was conducted with an anti‐immunoglobin horseradish peroxidase antibody (Beyotime Biotechnology, China) and detected using enhanced chemiluminescence (BIO‐RAD, USA). The densities of bands were assessed using Image J. As a control, GAPDH levels were evaluated in parallel.

### RT‐PCR and qRT‐PCR

Trizol Reagent (Vazyme Biotech, China) was used to extract total RNA, and HiScript II Q RT SuperMix for qPCR (Vazyme, China) was used to reverse transcribe the RNA. For qRT‐PCR, AceQ qPCR SYBR Green Master Mix (Vazyme, China) was used. Incubation of samples was performed at 37°C for 30 min with 5 U µg^−1^ of RNase R (Epicentre Technologies, USA). gDNA was isolated using genomic DNA isolation kits (TIANGEN Biotech, China). GAPDH was used to normalize circRNA and mRNA levels. The primer sequences were shown in Table S7 (Supporting Information).

### Cell Culture And Transfection

THP‐1, A549, and H1703 cells were purchased from the Shanghai Institute of Cell Biology, Chinese Academy of Sciences (Shanghai, China). Cell lines were cultured at 37°C with 5% CO_2_ in 10% fetal bovine serum (Shanghai, China) with RPMI 1640 medium containing 100 U ml^−1^ penicillin and 100 mg ml^−1^ streptomycin (Gibco, USA). Treatment with 100 ng ml^−1^ phorbol myristate acetate (PMA) (Sigma, USA) for 24 h induced the differentiation of THP‐1 cells into macrophages (THP‐M).^[^ ^61^ ^]^ THP‐M were induced in the M2 phenotype in the presence of 10 ng mL^−1^ interleukin‐4 (IL‐4) for 48 h. CSE was obtained as described previously.^[^ ^42^ ^]^ Briefly, in RPMI‐1640 medium, smoke from 3R4F research cigarettes was collected using a vacuum pump. CSE at various concentrations was applied to THP‐M for 48 h. circEML4 siRNA, ALKBH5 siRNA, and an siRNA con were acquired from RiboBio (Guanzhou, China). Cells were transfected with Lipofectamine 2000 (Invitrogen, USA) according to the manufacturer's instructions. The siRNA sequences were indicated in Table S8 (Supporting Information). A549 and H1703 cells (1 × 10^6^) were exposed to EVs (4 µg) from THP‐M or CSE‐THP‐M for 24 h.

### Flow Cytometry

Fc receptors in all samples were blocked with TruStain fcX (Biolegend, USA). THP‐M treated with various concentrations of CSE or IL‐4 were incubated with FITC‐conjugated anti‐human CD11b (BioLegend, USA) and APC‐conjugated anti‐human CD206 (Biolegend, USA). FlowJo (FlowJo LLC, USA) was used to test samples obtained from the FACSCanto (BD Biosciences, USA). Cell distribution was expressed as a percentage.

### CCK8 Assay

NSCLC cells (2 × 10^3^ per well) were seeded in 96‐well plates after transfection or treatment. To these cells, CCK8 solution (Dojindo, Japan) was added, and the preparations were incubated for 1 h at 37°C with 5% CO_2_. The cells were counted at selected time points. The absorbance was measured at 450 nm.

### Transwell Assay

Following treatment, NSCLC cells were digested, washed with PBS, and suspended in serum‐free RPMI‐1640 medium. Filters (8‐µm pore, Corning, USA) and Matrigel (BD Biosciences, USA) were used. Upper chambers were filled with serum‐free RPMI‐1640 containing NSCLC cells (5 × 10^4^/100 µL). To the lower chambers, 600 µL of RPMI‐1640 with 20% FBS was added. After 24 h, non‐migratory or non‐invasive cells were removed. Invading cells were fixed, stained, and counted.

### Colony Formation Assay

After being seeded into 6‐well plates with 5 × 10^2^ NSCLC cells, cells were cultured for 10 days. Images were taken after the cells were washed with PBS, fixed with 4% paraformaldehyde for 20 min, and stained with 0.1% crystal violet solution for 10 min.

### 5‐Ethynyl‐2′‐deoxyuridine (EdU) Staining

Incubation with RPMI‐1640 containing 50 µm EdU (RiboBio, China) was at 37°C with 5% CO_2_ for 2 h. PBS was used for washing the cells twice, 4% paraformaldehyde was applied for 30 min, and glycine was added for neutralizing and 0.5% Triton X‐100 for permeabilizing. Cells were incubated for 30 min at room temperature after washing with PBS, and then 100 µL of Apollo dye was added to each well. For the next 30 min, 100 µL of Hoechst 33 342 was added, and cells were visualized under a fluorescence microscope.

### Isolation of EVs

EVs were collected by density gradient ultracentrifugation as described previously.^[^ ^62^ ^]^ THP‐M culture medium was centrifuged at 800 × g for 5 min, followed by centrifugation at 2000 × g for 10 min to eliminate cellular debris. The supernatant was then passed through a 0.22‐µm PVDF filter (Millipore, USA) and centrifuged at 100 000 × g for 90 min at 4°C. The supernatant was discarded, and the pellets were washed with PBS and used to suspend the pellets. EVs were isolated from plasma of patients with NSCLC by use of Minute Hi‐Efficiency Exosome Precipitation Reagent (Invent Biotechnologies, USA). For removing large debris, 1‐mL plasma samples were centrifuged for 10 min at 2000 × g. Two volumes of EVs precipitation reagent were added to the supernatant, followed by 1 h of incubation. Purified EVs were obtained by centrifugation of the sample for 30 min at 10 000 × g, followed by centrifugation at 100 000 × g for 70 min. The size distribution and concentration of EVs were determined with a ZetaView particle tracker from ParticleMetrix (Germany). The shape of EVs was assessed with a transmission electron microscope (Tokyo, Japan). CD63, Alix, and TSG101 served as markers of EVs; GM130 served as a marker of cells. The concentrations of EVs were quantified with BCA protein assay kit (Beyotime Biotechnology, China).

### Actinomycin D Treatment

Seeded cells were cultured in 6‐well plates to 70%–80% confluency. The medium was supplemented with 2 µg ml^−1^ actinomycin D (Sigma, USA). After actinomycin D treatment, RNA was extracted at 0, 4, 8, and 24 h. qRT‐PCR was used to detect the expression of circRNA and mRNA.

### Fluorescence in Situ Hybridization (FISH)

Fluorescent In Situ Hybridization kits (Ribobio, China) were used for the FISH analysis. The cells were fixed in 4% paraformaldehyde for 10 min at room temperature. Two washes with PBS were followed by 5 min of exposure to 0.5% Triton X‐100 at 4°C. After pre‐hybridization for 30 min at 37°C, the samples were hybridized with circRNA probes (GenePharma, China) for 12–16 h at 37°C. There were three washes with a 42°C solution containing 0.1% Tween‐20 and 4 × saline sodium citrate (SSC), followed by washes with 2 × SSC and 1 × SSC. The samples were then stained with 4′,6‐diamidino‐2‐phenylindole (DAPI) for 5 min. Sections of NSCLC tissues (5‐µm thickness), cut from paraffin‐embedded blocks, were hybridized and analyzed. A microscope was used to image the samples (Zeiss, Germany). For sequences used for FISH, see Table S9 (Supporting Information).

### Animal Experiments

All animal experiments were approved by the Animal Protection and Use Committee of Nanjing Medical University (no. IACUC‐2105048). Female NOD/SCID mice at 6 weeks of age (Gem Pharmatech, China) were used to explore the effect of CSE‐induced macrophages on subcutaneous tumorigenesis of NSCLC cells. Female Balb/c nude mice at 4 weeks of age were obtained from the Experimental Animal Center of Nanjing Medical University, housed under specific pathogen‐free conditions, and provided with standard food. A549 cells (5 × 10^6^) were subcutaneously transplanted into nude mice. Tumor size was assessed every 7 days. EVs (10 µg) were injected subcutaneously into mice every 3 days. The mice were sacrificed, tumors were removed, and their weights were recorded in the fourth week. In addition, the nude mice were injected with A549 cells (2 × 10^6^) through the tail vein, and EVs (10 µg) were injected into mice via the tail vein every 3 days. The bilateral lung tissues were resected and stained with hematoxylin‐eosin.

### RNA Immunoprecipitation (RIP)

RIP was performed following the instructions of the manufacturer with Magna RIP RNA‐Binding Protein Immunoprecipitation Kits (Millipore, USA). Magnetic beads were coated with 5 µg of an antibody, including anti‐IgG, anti‐METTL3, anti‐METTL14, anti‐WTAP, anti‐ALKBH5, or anti‐FTO for 30 min at room temperature. Lysates from 2 × 10^7^ cells were incubated overnight with antibody‐coated magnetic beads. After washing the magnetic beads‐protein‐RNA complexes with wash buffer, they were incubated with proteinase K digestion buffer at 55°C for 30 min. Purified RNAs were analyzed by qRT‐PCR.

### m6A‐seq and RNA‐seq

mRNA Isolation Kits (Promega, USA) were used to isolate intact mRNA from total RNA samples of A549 cells transfected with ALKBH5 siRNA or siRNA con. Incubation at 94°C for 5 min resulted in chemical fragmentation of the mRNA into 200‐nucleotide fragments, which were analyzed using an Agilent 2100 Bioanalyzer (Agilent, USA). Then, with the m6A‐antibody, methylated mRNAs were immunoprecipitated. Another transcriptome sequencing library was constructed as a control. The eluted RNA and MeRIPed RNA were analyzed by deep sequencing at LC‐BIO Biotechnology (Hangzhou, China) using an Illumina Novaseq 6000 platform. The package exomePeak used mapped reads from immunoprecipitations (IPs) and input libraries to identify m6A peaks. The m6A peak was visualized using the UCSC genome browser or IGV software. *De novo* and known motif findings were accomplished with HOMER, followed by localization of the motif by Perl scripts. Using StringTie, fragments per kilobase of transcript per million was calculated for all mRNAs in input libraries. GSEA was conducted using the R package clusterProfiler.

### m6A Dot Blot Assay

The mRNA concentration obtained from the total cellular RNA was determined using a NanoDrop 2000 instrument, and the mRNA was diluted to various concentrations with RNase‐free water. Serially diluted mRNA sample were denatured at 95 °C for 3 min and chilled on ice. The nucleic acid was transferred onto a Hybond‐N^+^ membrane (Beyotime Biotechnology, China) through a spotter and then 2400 UV cross‐linked to the membrane. After washing, the membrane was incubated with m6A antibody in 5% nonfat milk at 4°C overnight. The following day, an additional incubation of 1 h was conducted with HRP‐conjugated secondary antibodies (ZSGB‐BIO, China) and measured with ECL reagents (BIO‐RAD, USA). Methylene blue in 0.3 m sodium acetate staining was used as a loading control.

### Methylated RNA Immunoprecipitation (meRIP)‐qPCR

RNA was extracted from NSCLC cells using Trizol Reagent (Vazyme Biotech, China). Dynabeads mRNA Purification Kits (Invitrogen, USA) were used to purify mRNA. A portion of RNA was used as an input control. The sample was incubated overnight at 4°C in immunoprecipitation buffer containing m6A‐antibody (Synaptic Systems, Germany). Then, immunoprecipitation and elution of samples containing m6A RNA were performed. By normalizing to the input, the m6A enrichment in each sample was determined by qRT–PCR.

### Cytoplasmic and Nuclear Protein Extraction

To extract nuclear and cytoplasmic proteins, a commercial kit (Beyotime Biotechnology, China) was used as directed by the manufacturer. Briefly, cells were harvested and suspended in PMSF‐containing solution A. Following a 10‐min ice bath, the cell lysates were combined with 10 µL of solution B and placed on ice for 1 min. After centrifugation at 15 000 rpm, cytoplasmic proteins were obtained from the supernatant. Nuclear protein extraction reagent with PMSF (50 µL) was added to the precipitate. The supernatant was centrifuged at 12 000 rpm to obtain the nuclear proteins.

### Dual‐Luciferase Reporter Assays

A dual‐luciferase reporter kit (Promega, USA) was used to assess luciferase activity. Firefly and Renilla luciferases were included in the reporter plasmid created using the GeneChem pGL3 expression vector. In the mutant SOCS2 reporter plasmid, adenine (A) bases were replaced with guanine (G) bases. Luciferase activity was measured using the Duo‐Lite Luciferase Assay System (Vazyme Biotech, China), as directed by the manufacturer.

### RNA Pull‐Down Assay

RNA pull‐down was accomplished using a kit (BersinBio, China). Biotinylated circEML4 probes (4 µg) were incubated with streptavidin‐coated magnetic beads for 30 min at 25°C. Then, nucleic acids were removed. Incubation of the lysate with streptavidin‐coated magnetic beads and a biotinylated probe at 4°C was performed. Beads‐probe‐protein complexes were washed 4 times with NT2 buffer. After washing the magnetic beads five times, Western blots were used to determine if binding proteins were enriched. In addition, acquired proteins were stained using Fast Silver Stain Kits (Beyotime, China) following an RNA pulldown assay. SDS‐PAGE samples were analyzed by mass spectrometry (MS). For the sequences used for RNA pull‐down, see Table S10 (Supporting Information).

### Statistical Analysis

Data analyses were conducted with 22.0 SPSS (Chicago, IL, USA). Values were expressed as means ± standard deviation. For categorical outcomes, logistic regression models were used. Student's *t*‐test, chi‐square or Fisher's exact test, and one‐way ANOVA were used for the statistical analysis. The least significant difference (LSD) was used when comparing groups. Cigarette smoking levels were correlated with circEML4, CD206, SOCS2 using Spearman correlation analysis. *p* < 0.05 was considered statistically significant.

## Conflict of Interest

The authors declare no conflict of interest.

## Author Contributions

C.C., P.W., and Y.Y. contributed equally to this study. Q.L., H.W., and C.C. developed the hypothesis. C.C., P.W., Y.Y., X.D., H.X., and L.L. performed the vitro experiments. P.W., Y.Y., and X.D. conducted vivo experiments. C.C. and Y.Y. conducted the bioinformatics analyses. X.D., J.L., and H.W. were responsible for clinical samples and clinical data collection. Q.L. and H.W. revised the manuscript.

## Supporting information

Supporting Information

## Data Availability

The data that support the findings of this study are available from the corresponding author upon reasonable request.
