# Supplementary material for: Smoking‐Induced M2‐TAMs, via circEML4 in EVs, Promote the Progression of NSCLC through ALKBH5‐Regulated m6A Modification of SOCS2 in NSCLC Cells
Source: Adv Sci (Weinh). 2023 May 28;10(22):2300953. doi: 10.1002/advs.202300953 (PMC10401136; doi:10.1002/advs.202300953)
Supplement: Supplementary file 1 — Supporting Information [file ADVS-10-2300953-s001.pdf]

ADVANCED SCIENCE
Open Access

Supporting Information

for *Adv. Sci.*, DOI 10.1002/advs.202300953

Smoking-Induced M2-TAMs, via circEML4 in EVs, Promote the Progression of NSCLC through ALKBH5-Regulated m6A Modification of SOCS2 in NSCLC Cells

Cheng Cheng, Peiwen Wang, Yi Yang, Xuan Du, Haibo Xia, Jinyuan Liu, Lu Lu, Hao Wu and Qizhan Liu**

Supplementary figures and figure legends

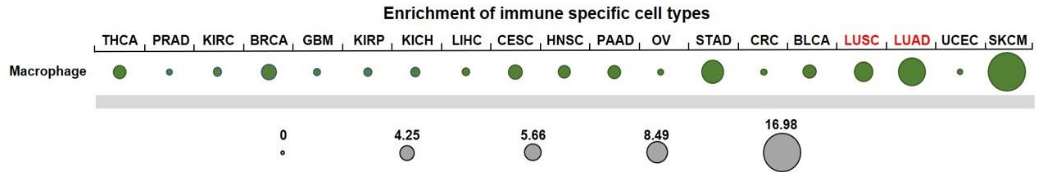

Figure S1. Macrophage distribution in the TME evaluated using the Cancer Immunome Atlas (https://tcia.at/). The results of the ssGSEA are presented by a bubble plot. Size of circles shows the size percentage of patients with NES >0 and q value (FDR) < 0.1.

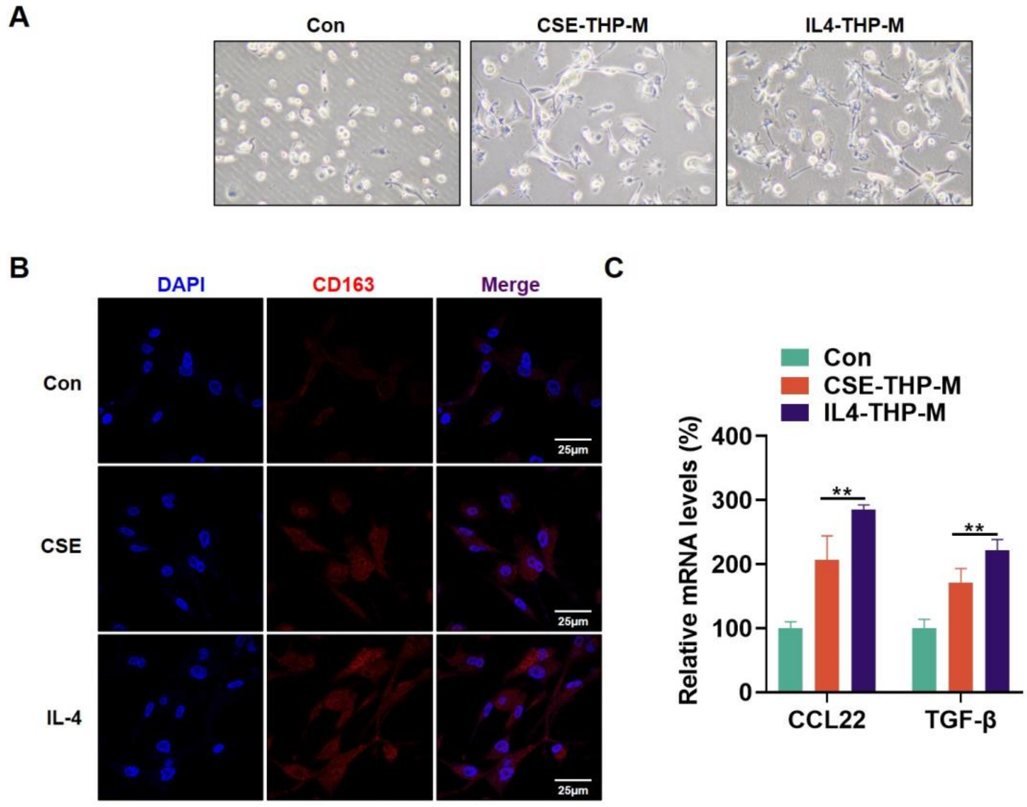

Figure S2. CSE induces M2 polarization in THP-M.

THP-M were exposed to 4% CSE for 48 h; IL-4 was used as a positive control. (A) THP-M treated with IL-4 and CSE showed morphological changes. (B) Representative images of CD163 immunofluorescence as determined by confocal microscopy for

THP-M. DAPI labels nuclei (blue). Scale bar, 25 μm. (C) The mRNA levels of CCL22 and TGF-β in THP-M were measured by qRT-PCR. Three independent experiments were conducted. All data represent means ± SD. *$P < 0.05$; **$P < 0.01$.

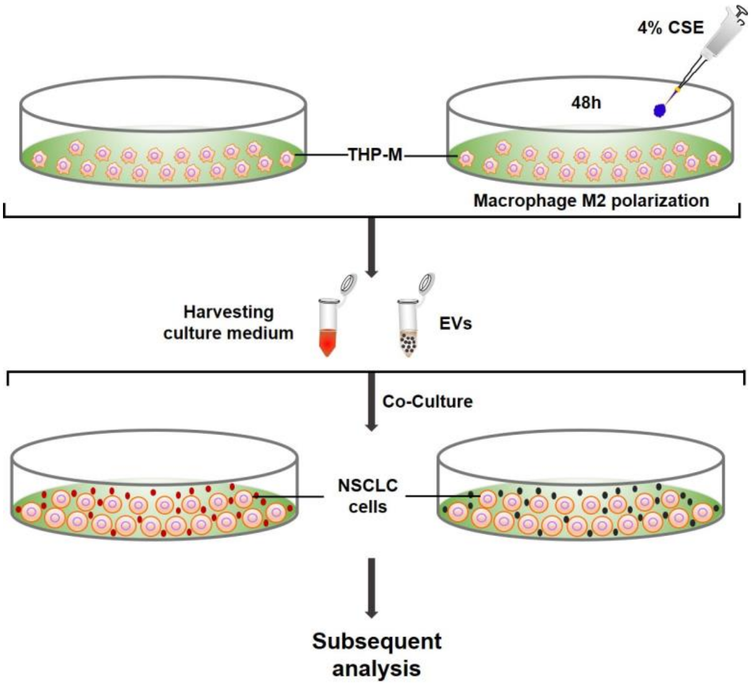

Figure S3. Schematic description of the experimental design.

CM or EVs from THP-M treated with CSE (4%) for 48 h. The NSCLC cells were treated with CM and EVs for 24 h. Cells were treated with CM or EVs every 3 days for the CCK8 and colony formation experiments.

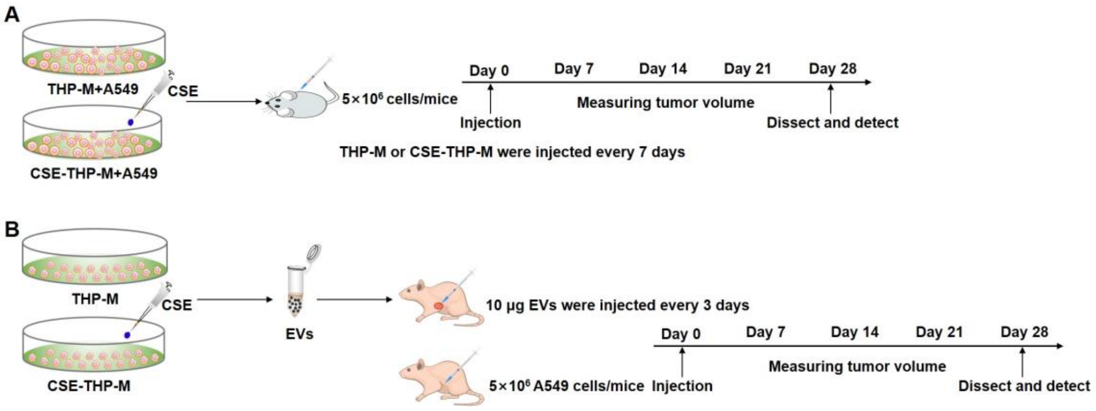

Figure S4. Xenograft mice model.

THP-M were exposed to 4% CSE for 48 h. (A) Schematic illustration showing that THP-M or CSE-THP-M were mixed with A549 cells and injected into NOD/SCID mice. (B) EVs (10 μg) were injected intratumorally every 3 days into the tumor centers of mice in each group (randomly divided).

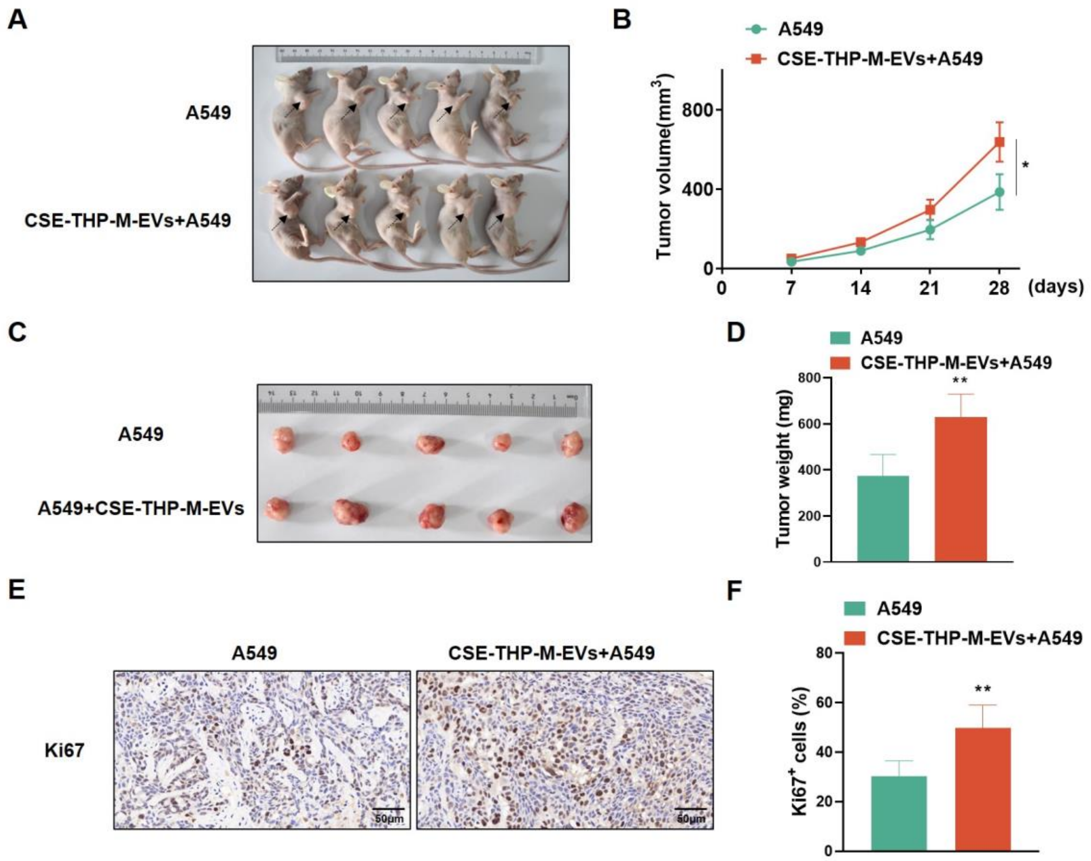

Figure S5. The roles of EVs from CSE-THP-M on NSCLC cells *in vivo*

(A) Tumors of the subcutaneous layer were observed for two groups. (The arrows point to tumor xenografts.) (B) Tumor volume summary for mice, measured every 7 days. (C) Images of xenograft tumors harvested from nude mice. (D) Tumor weights (n = 5) were recorded. (E) IHC staining for Ki67 was assessed (n = 5), and (F) measurement of Ki67-positive signals in tumor tissues harvested from nude mice (n = 5 randomly selected fields per group). Scale bar, 50 μm. Three independent experiments were

conducted. All data represent means ± SD. *P < 0.05; **P < 0.01.

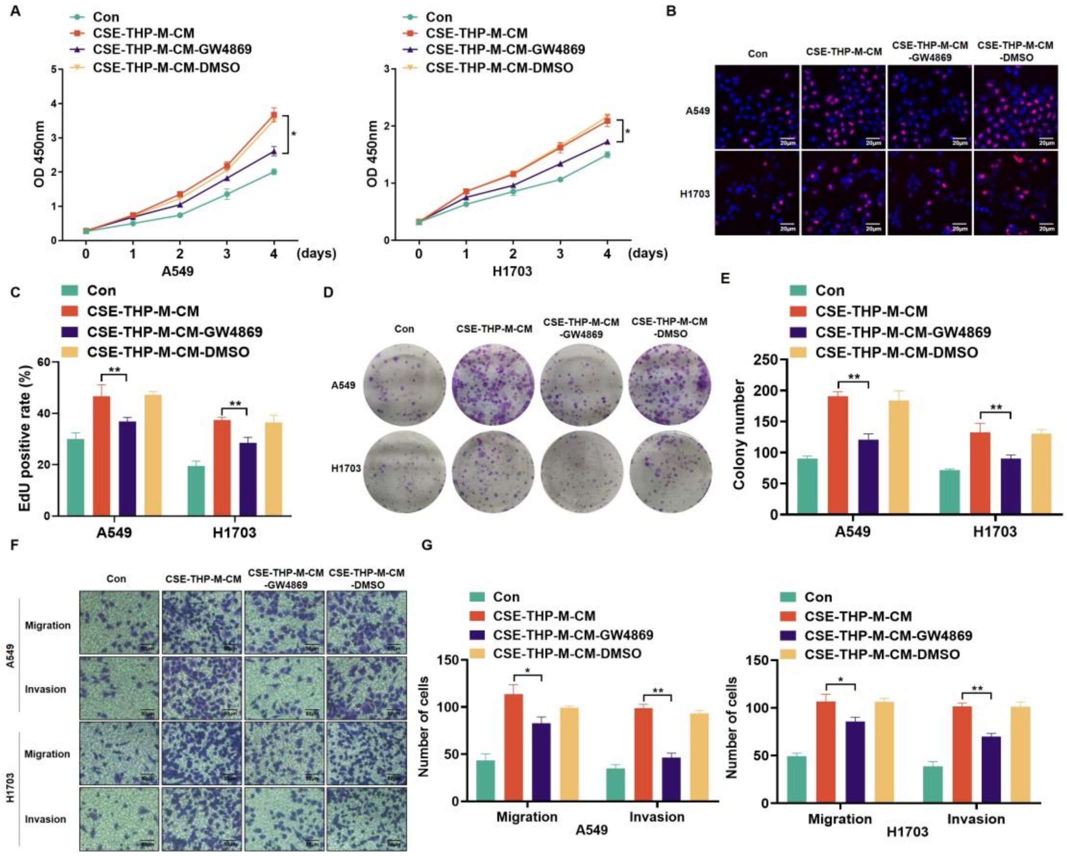

Figure S6. Role of inhibition of secretion of EVs from CSE-induced M2 macrophages on proliferation, migration, and invasion of NSCLC cells.

THP-M were exposed to 4% CSE for 48 h. The proliferation of A549 and H1703 cells was measured by (A) CCK-8 assays. (B) EdU assays and (C) EdU-positive rates were determined. (D) Colony formation assays were performed, and (E) colony formation assays were conducted for A549 and H1703 cells. (F) Representative images of Transwell assays, and (G) the migration and invasion were assessed for A549 and H1703 cells. Three independent experiments were conducted. All data represent means ± SD. *P < 0.05; **P < 0.01.

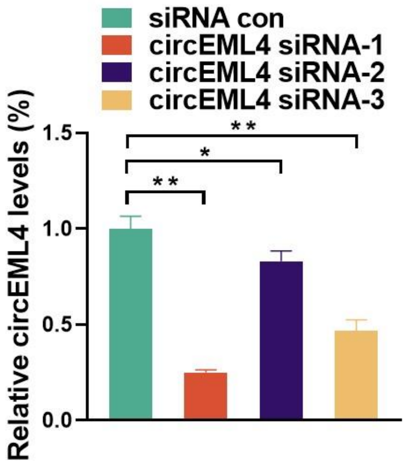

Figure S7. Validation of circEML4 knockdown in THP-M.

THP-M were exposed to siRNA con or to circEML4 siRNA for 24 h.

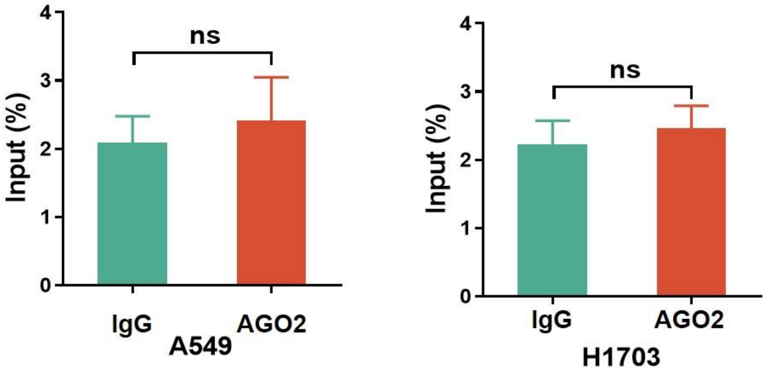

Figure S8. The binding capacity of circEML4 to AGO2 in NSCLC cells was assessed.

With extracts from A549 and H1703 cells, RIP experiments were performed with an antibody against AGO2. Three independent experiments were conducted. All data represent means ± SD. ns, not significant. *P < 0.05; **P < 0.01.

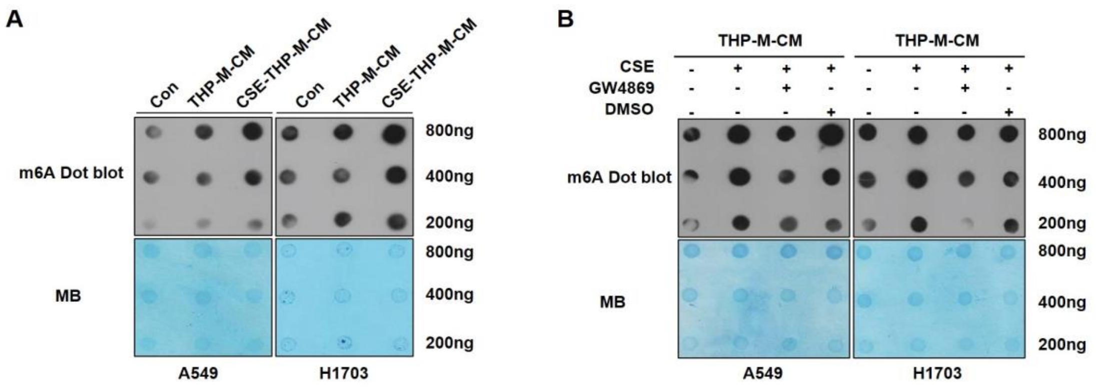

Figure S9. The role of inhibition of EVs secretion from CSE-induced M2 macrophages on m6A contents of NSCLC cells.

THP-M were exposed to 4% CSE for 48 h. A549 and H1703 cells were exposed to THP-M-CM or CSE-THP-M-CM for 24 h after GW4869 or DMSO co-incubation with THP-M. (A) and (B) RNA m6A dot blot assays were used to detect the m6A contents of total mRNA. Methylene blue staining was used to determine the loading control.

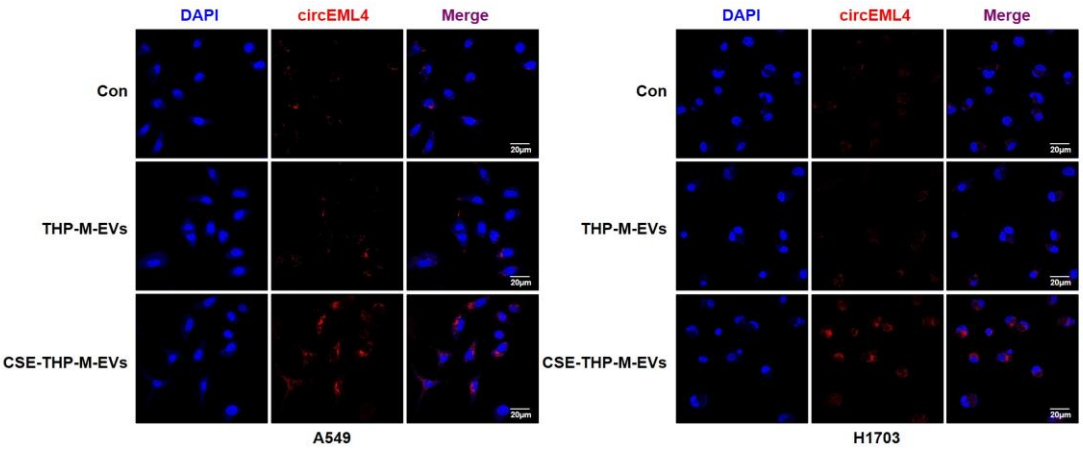

Figure S10. The uptake of circEML4 in EVs from CSE-induced M2 macrophages by NSCLC cells is increased.

THP-M were exposed to 4% CSE for 48 h. A549 and H1703 cells were exposed to THP-M-EVs or CSE-THP-M-EVs for 24 h. RNA fluorescence in situ hybridization for circEML4 (red) in A549 and H1703 cells. Nuclei were stained with DAPI (blue). Scale bar, 25 μm.

A

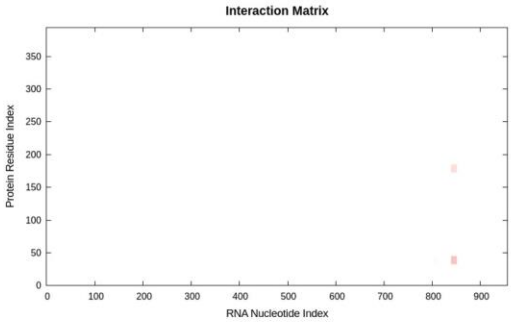

B

#	Protein region	RNA region	Interaction Propensity	Discriminative Power	Normalized Score
1	26-77	831-882	7.24	24	2.76
2	169-220	831-882	6.55	22	2.55
3	26-77	801-852	5.84	22	2.34
4	26-77	376-427	5.81	22	2.33
5	26-77	826-877	5.39	22	2.21
6	176-227	831-882	5.13	22	2.13
7	169-220	826-877	5.00	20	2.09
8	169-220	376-427	4.87	20	2.05
9	119-170	831-882	4.81	20	2.03
10	169-220	801-852	4.76	20	2.02
11	69-120	831-882	4.41	20	1.91
12	126-177	831-882	4.41	20	1.91
13	276-327	831-882	4.33	20	1.89
14	94-145	831-882	4.28	20	1.87
15	51-102	131-182	4.21	20	1.85
16	26-77	806-857	4.11	20	1.82
17	51-102	256-307	3.94	20	1.77
18	44-95	256-307	3.93	20	1.77
19	69-120	801-852	3.92	20	1.76
20	176-227	801-852	3.92	20	1.76

C

CCTCTACAACCCCACGTCAGAGTGTGGGATTCTGTTACTCTATCCACACTGCAGATTAT
TGGACTTGGCACTTTTGAGCGTGGAGTAGGATGCCTGGATTTTTCAAAAGCAGATTCA
GGTGTTCATTTATGTATTATTGATGACTCCAATGAGCATATGCTTACTGTATGGGACTGGC
AGAAGAAAGCAAAAGGAGCAGAAATAAAGACAACAAATGAAGTTGTTTTGGCTGTGG
AGTTTCACCCAACAGATGCAAATACCATAATTACATGCGGTAAATCTCATATTTTCTTCT
GGACCTGGAGCGGCAATTCACTAACAAGAAAACAGGGAATTTTTGGGAAATATGAAA
AGCCAAAATTTGTGCAGTGTTTAGCATTCTTGGGGAATGGAGATGTTCTTACTGGAGAC
TCAGGTGGAGTCATGCTTATATGGAGCAAAACTACTGTAGAGCCCACACCTGGGAAAG
GACCTAAAGGTGTATATCAAATCAGCAAACAAATCAAAGCTCATGATGGCAGTGTGTTC
ACACTTTGTCAGATGAGAAATGGGATGTTATTAACTGGAGGAGGGAAAGACAGAAAA
ATAATTCTGTGGGATCATGATCTGAATCCTGAAAGAGAAATAGAGGTTCCTGATCAGTA
TGGCACAATCAGAGCTGTAGCAGAAGGAAAGGCAGATCAATTTTTAGTAGGCACATCA
CGAAACTTTATTTTACGAGGAACATTTAATGATGGCTTCCAAATAGAAGTACAGGGTCA
TACAGATGAGCTTTGGGGTCTTGCCACACATCCCTTCAAAGATTTGCTCTTGACATGTG
CTCAGGACAGGCAGGTGTGCCTGTGGAACTCAATGGAACACAGGCTGGAATGGACCA
GGCTGGTAGATGAACCAGGACACTGTGCAGATTTTCATCCAAGTGGCACAGTGGTGGC
CATAGGAACGCACTCAGGCAG

Figure S11. Bioinformatics predicts the binding of circEML4 to ALKBH5 protein. (A) and (B) Binding sites between circEML4 and ALKBH5 predicted by the catRAPID website (http://service.tartaglialab.com/page/catrapid_group). (C) The sequence of mutated circEML4. The binding sequence of circEML4 with ALKBH5 (Red) was predicted by CatRAPID.

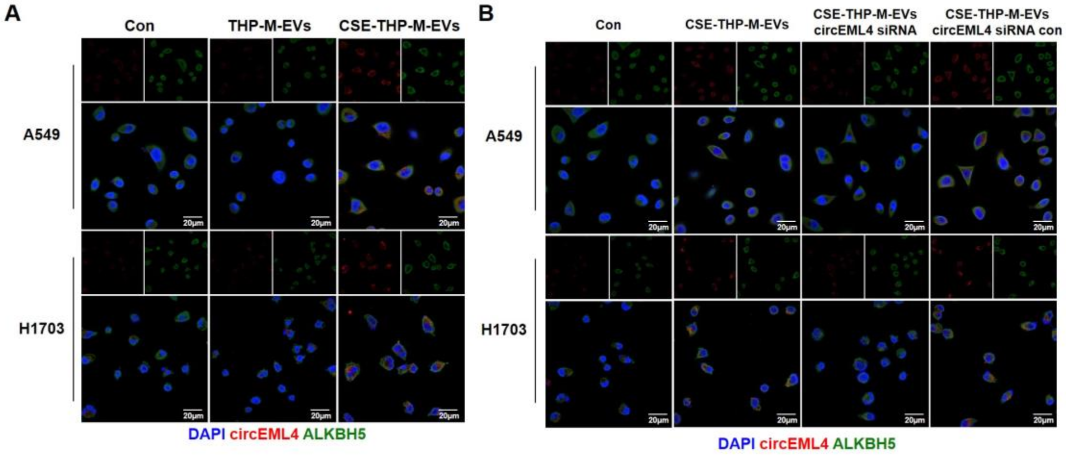

Figure S12. The cellular localization of circEML4 and ALKBH5 in NSCLC cells.

CSE-THP-M were treated with circEML4 siRNA or siRNA con before co-incubation with A549 or H1703 cells. (A) and (B) Immunofluorescence evaluation of circEML4 (red) and ALKBH5 (green) co-localization in A549 and H1703 cells. Nuclei were stained with DAPI (blue). Scale bar, 20 μm.

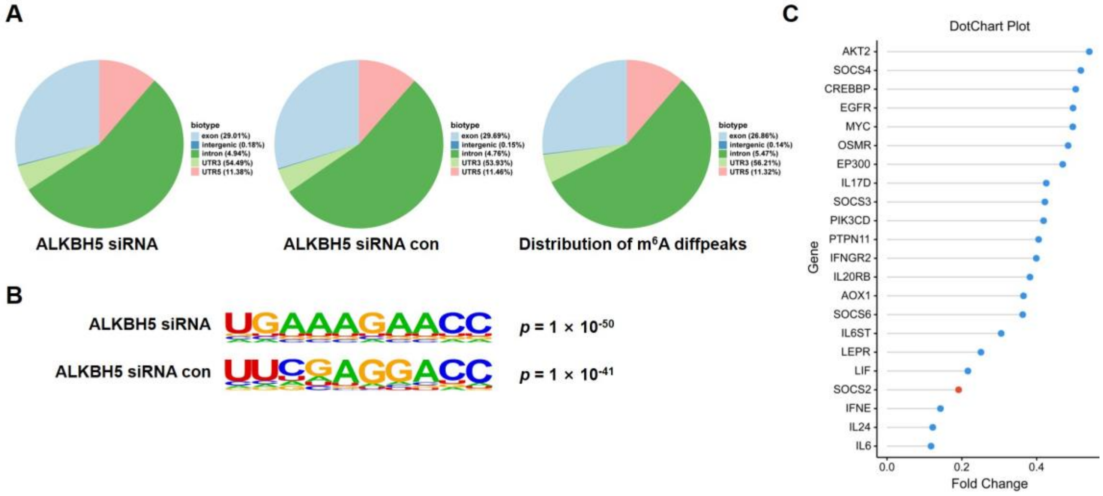

Figure S13. Analysis of m6A-seq and RNA-seq of A549 cells.

(A) Density distribution of m6A peaks across mRNA transcripts in A549 cells with or without ALKBH5 siRNA. (B) The predominant consensus motif was measured by m6A-seq. (C) Fold change of the JAK-STAT pathway by GSEA enrichment analysis at mRNA levels.

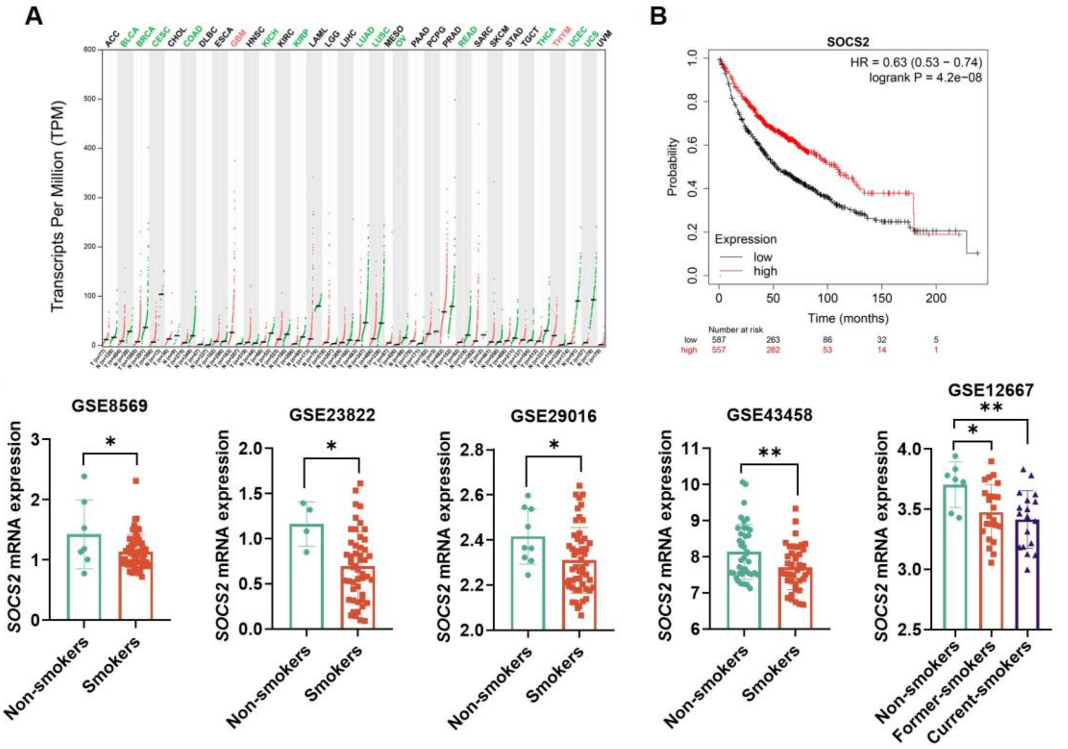

Figure S14. Expression and prognostic value of SOCS2 in lung cancer.

(A) Expression of SOCS2 in tumor and normal samples based on TCGA dataset. (B) Validation of the prognostic value of SOCS2 in lung cancer using the KM Plotter tool. (C) Expression of SOCS2 for smoking or never-smoking lung cancer patients based on the GEO dataset. Outliers were eliminated during the analysis. Z-score was used for identifying outliers and calculated as follows: $z = \frac{x-\bar{x}}{\sigma}$, $z = \frac{x-\bar{x}}{\sigma}$ where x was the value to be normalized in the data, $\bar{x}$ represented the mean of the data, and σ was the standard deviation of the data. Data were considered as an outlier if the Z-score was greater than or less than 2 or -2, respectively. All data represent means ± SD. **P* < 0.05; ***P* < 0.01.

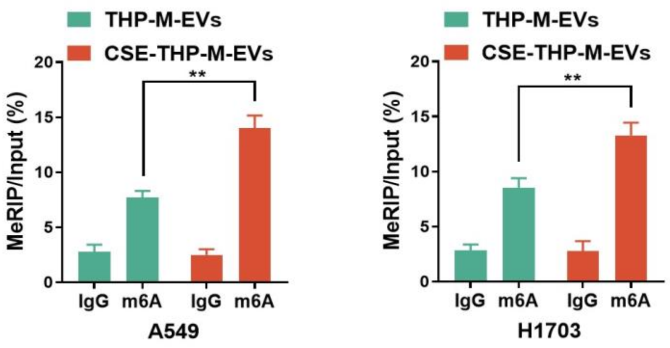

Figure S15. Release of EVs derived from CSE-induced M2 macrophages increases m6A levels for SOCS2 in NSCLC cells.

THP-M were exposed to 4% CSE for 48 h. A549 and H1703 cells were exposed to THP-M-EVs or CSE-THP-M-EVs for 24 h. MeRIP-qPCR determinations for m6A enrichment on SOCS2 in A549 and H1703 cells. Three independent experiments were conducted. All data represent mean ± SD. *P < 0.05; **P < 0.01.

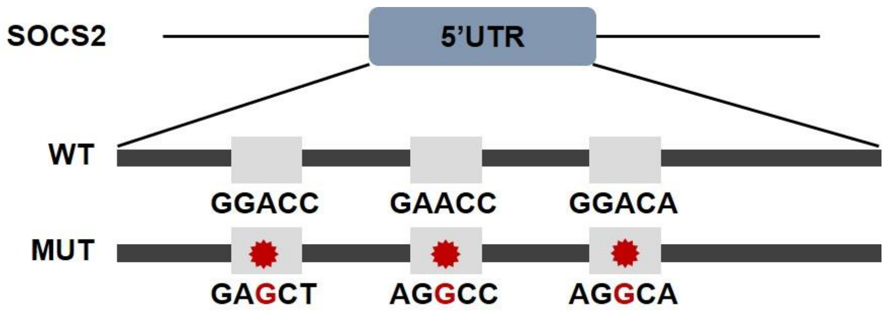

Figure S16. Schematic depiction of mutations of the m6A site in SOCS2.

The WT or m6A consensus sequence mutant SOCS2 5'UTR was fused with a firefly luciferase reporter. The m6A mutant sequence was constructed by replacing A with G.

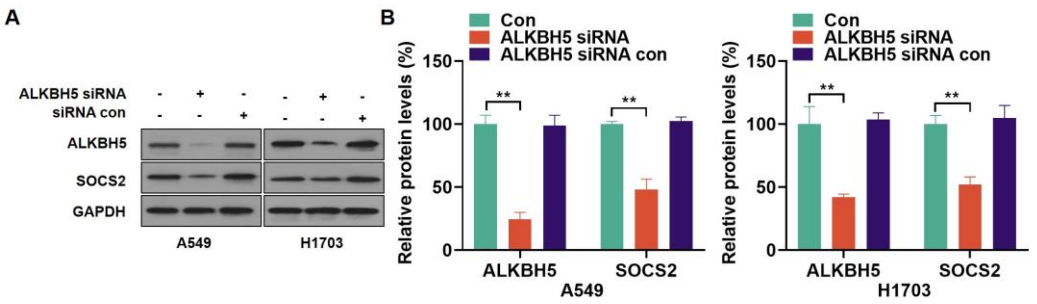

Figure S17. Regulation of SOCS2 protein levels by ALKBH5.

A549 and H1703 cells were treated with ALKBH5 siRNA or siRNA con for 24 h. (A) Western blots were performed, and (B) relative protein levels of ALKBH5 and SOCS2 were determined. Three independent experiments were conducted. All data represent means ± SD. **P* < 0.05; ***P* < 0.01.

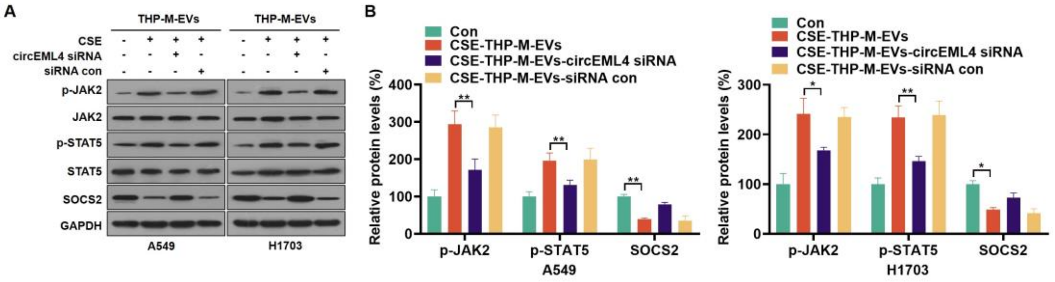

Figure S18. circEML4 in EVs from CSE-induced M2 macrophages regulates the SOCS2/JAK-STAT pathway in NSCLC cells.

THP-M were treated with circEML4 siRNA or siRNA con for 24 h, and then exposed to CSE (4%) for 48 h. A549 and H1703 cells were exposed to THP-M-EVs or CSE-THP-M-EVs for 24 h. (A) Western blots were performed, and (B) relative protein levels of p-JAK2, p-STAT5, and SOCS2 were determined. Three independent experiments were conducted. All data represent means ± SD. **P* < 0.05; ***P* < 0.01.

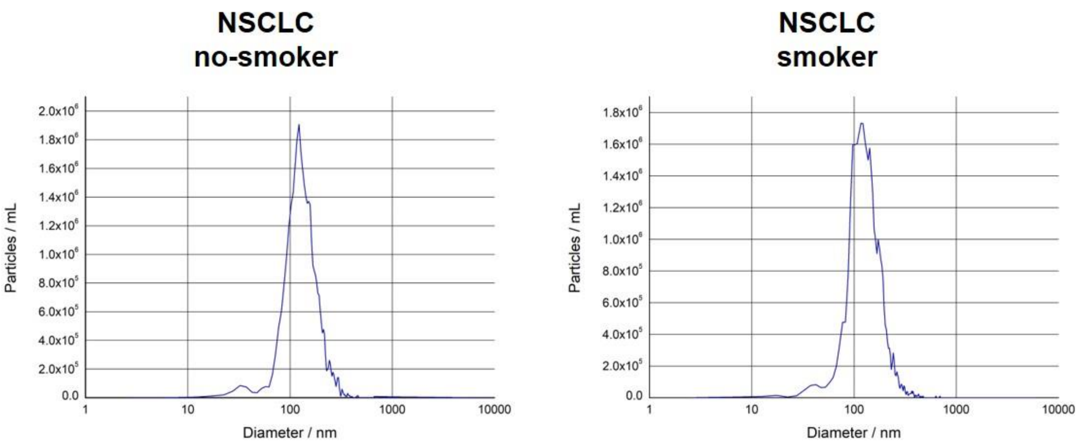

Figure S19. Particle number and size analysis of EVs in plasma of NSCLC patients.

Supplementary tables

Table S1. Clinical characteristics of NSCLC patients.

**Characteristic**	**Non-Smokers (n=48)**	**Smokers (n=70)**	***P* value**
Sex			0.513
Male, (%)	28(58.3%)	45 (64.3%)	
Female, (%)	20 (41.7%)	25 (35.7%)	
Age(years), Mean ± SD	60.0±11.6	62.1±7.9	0.263
Package year, Mean ± SD		40.6±24.0	NA
Histology type			
adenocarcinoma	24 (50.0%)	27 (38.6 %)	0.354
squamous carcinoma	22 (45.8%)	36 (51.4%)	
others	2 (4.2%)	7 (10.0%)	
Tumor grade			
T1	40 (83.3%)	35 (50.0%)	<0.001
T2-T4	8 (16.7%)	35 (50.0%)	
Lymphatic metastasis			
negative	29 (60.4%)	25(35.7%)	0.008
positive	19 (39.6%)	45 (64.3%)	
Distant metastasis			
no	40 (83.3%)	49 (70.0%)	0.098
yes	8 (16.7%)	21 (30.0%)	
TNM stage			
0-II	39 (81.2%)	37 (52.9%)	0.002
III-IV	9 (18.8%)	33 (47.1%)	
Maximum tumor diameter			<0.001
≤3cm	45 (93.8%)	44 (62.9%)	
>3cm	3 (6.2%)	26 (37.1%)	

$P < 0.05$ was considered statistically significant.

Table S2. 13 common up-regulated circRNAs obtained from GSE112214 and GSE158695

probeID	Annotations					GSE112214		GSE158695	
	CircRNA	Alias	Position	Strand	Gene Symbol	FC	P-value	FC	P-value
ASCRP003393	hsa_circRNA_103089	hsa_circ_0008253	chr20: 50342357-50346517	-	ATP9A	1.870	<0.001	6.296	0.007
ASCRP000991	hsa_circRNA_100616	hsa_circ_0004606	chr10: 72462067-72468534	+	ADAMTS14	1.583	0.001	2.200	0.014
ASCRP000922	hsa_circRNA_100542	hsa_circ_0017639	chr10: 7290509-7327916	-	SFMBT2	1.954	0.001	5.563	0.034
ASCRP003014	hsa_circRNA_102702	hsa_circ_0054243	chr2: 42513408-42531691	+	EML4	1.670	0.001	1.562	0.007
ASCRP000874	hsa_circRNA_100491	hsa_circ_0003314	chr1: 233353644-233363117	-	PCNXL2	1.584	0.001	1.984	0.010
ASCRP004411	hsa_circRNA_104138	hsa_circ_0077086	chr6: 76357446-76380436	+	SENP6	1.680	0.005	2.102	0.013
ASCRP002200	hsa_circRNA_101857	hsa_circ_0040148	chr16: 70178323-70180131	+	PDPR	1.830	0.007	1.589	0.004
ASCRP002347	hsa_circRNA_102011	hsa_circ_0042521	chr17: 26512204-26512291	+	NLK	1.809	0.017	1.505	0.012
ASCRP001824	hsa_circRNA_101473	hsa_circ_0034447	chr15: 39876188-39876388	+	THBS1	1.730	0.019	2.369	0.003
ASCRP004150	hsa_circRNA_103862	hsa_circ_0072758	chr5: 68467096-68470236	+	CCNB1	1.622	0.021	2.688	0.008
ASCRP003427	hsa_circRNA_103123	hsa_circ_0002360	chr21: 36206706-36231875	-	RUNX1	2.143	0.023	2.472	0.006
ASCRP003013	hsa_circRNA_102701	hsa_circ_0054241	chr2: 42513408-42515462	+	EML4	1.514	0.026	1.785	0.021
ASCRP004228	hsa_circRNA_103948	hsa_circ_0003528	chr5: 134032815-134044578	+	SEC24A	1.633	0.037	1.878	0.002

Condition pairs: Tumorous tissue vs Nontumorous tissue

probeID: Represents probe name.

P-value: Calculated from paired t-test.

Annotations: Include circRNA, Alias, Position, strand, and GeneSymbol.

Table S3. List of top 10 candidates of circEML4-binding proteins that were identified by RNA pull-down and MS.

Accession	Gene Name	MW (kDa)	Unique Peptides	Coverage (%)
Q6P6C2	ALKBH5	44.2	20	54.1
Q96DH6	MSI2	35.2	5	21.3
P61923	COPZ1	20.2	5	32.8
P63173	RPL38	8.2	5	47.1
P46783	RPS10	18.9	5	29.1
Q9Y5K5	UCHL5	37.6	5	17.3
Q2TB90	HKDC1	102.5	4	6.2
P63241	EIF5A	16.8	4	18.8
Q01105	SET	33.5	4	16.6
O95819	MAP4K4	142.0	4	5.9

Table S4. GSEA enrichment analysis at m6A levels

ID	Description	Set size	Enrichment score	NES	P-value	P.adjust	q-value
hsa00650	Butanoate metabolism	10	0.719	1.662	0.009	0.031	0.018
hsa00770	Pantothenate and CoA biosynthesis	11	0.701	1.683	0.005	0.023	0.013
hsa05150	Staphylococcus aureus infection	13	0.683	1.739	0.006	0.025	0.014
hsa00982	Drug metabolism - cytochrome P450	11	0.681	1.634	0.011	0.034	0.020
hsa00051	Fructose and mannose metabolism	18	0.651	1.818	0.003	0.014	0.008
hsa05032	Morphine addiction	33	0.633	2.059	<0.001	0.001	0.001
hsa04727	GABAergic synapse	31	0.607	1.958	<0.001	0.004	0.002
hsa04610	Complement and coagulation cascades	28	0.584	1.814	0.002	0.012	0.007
hsa04974	Protein digestion and absorption	29	0.576	1.822	0.003	0.015	0.008
hsa01521	EGFR tyrosine kinase inhibitor resistance	53	0.572	2.064	<0.001	0.001	<0.001
hsa03020	RNA polymerase	22	0.572	1.693	0.007	0.028	0.016
hsa04724	Glutamatergic synapse	43	0.571	1.959	<0.001	0.003	0.002
hsa00970	Aminoacyl-tRNA biosynthesis	29	0.570	1.803	0.003	0.017	0.010
hsa04726	Serotonergic synapse	36	0.565	1.870	0.001	0.008	0.005
hsa04934	Cushing syndrome	67	0.564	2.119	<0.001	<0.001	<0.001
hsa05219	Bladder cancer	30	0.563	1.804	0.002	0.012	0.007
hsa04540	Gap junction	45	0.548	1.904	<0.001	0.004	0.002
hsa02010	ABC transporters	22	0.546	1.615	0.016	0.045	0.026
hsa04916	Melanogenesis	43	0.542	1.862	0.001	0.008	0.005
hsa04550	Signaling pathways regulating pluripotency of stem cells	74	0.538	2.054	<0.001	<0.001	<0.001
hsa04623	Cytosolic DNA-sensing pathway	30	0.537	1.722	0.005	0.021	0.012
hsa03440	Homologous recombination	31	0.531	1.714	0.006	0.025	0.014
hsa04371	Apelin signaling pathway	74	0.530	2.024	<0.001	<0.001	<0.001
hsa05226	Gastric cancer	76	0.529	2.045	<0.001	<0.001	<0.001
hsa04370	VEGF signaling pathway	39	0.529	1.781	0.003	0.016	0.009
hsa05225	Hepatocellular carcinoma	103	0.526	2.103	<0.001	<0.001	<0.001
hsa04072	Phospholipase D signaling pathway	80	0.525	2.046	<0.001	<0.001	<0.001
hsa05205	Proteoglycans in cancer	133	0.520	2.170	<0.001	<0.001	<0.001

hsa05224	Breast cancer	75	0.518	1.994	<0.001	0.001	<0.001
hsa05217	Basal cell carcinoma	31	0.516	1.667	0.010	0.033	0.019
hsa00280	Valine, leucine and isoleucine degradation	29	0.515	1.629	0.018	0.050	0.029
hsa05223	Non-small cell lung cancer	50	0.511	1.827	0.001	0.008	0.005
hsa05206	MicroRNAs in cancer	104	0.509	2.039	<0.001	<0.001	<0.001
hsa04725	Cholinergic synapse	49	0.508	1.801	0.002	0.012	0.007
hsa05221	Acute myeloid leukemia	37	0.504	1.683	0.009	0.031	0.018
hsa04713	Circadian entrainment	38	0.503	1.693	0.005	0.020	0.012
hsa04012	ErbB signaling pathway	62	0.501	1.861	0.001	0.006	0.003
hsa04730	Long-term depression	30	0.501	1.606	0.013	0.040	0.023
hsa04662	B cell receptor signaling pathway	38	0.497	1.672	0.006	0.024	0.014
hsa00310	Lysine degradation	40	0.494	1.671	0.006	0.024	0.014
hsa04014	Ras signaling pathway	114	0.492	2.011	<0.001	<0.001	<0.001
hsa04146	Peroxisome	45	0.489	1.701	0.003	0.017	0.010
hsa04115	p53 signaling pathway	46	0.485	1.701	0.004	0.020	0.012
hsa05231	Choline metabolism in cancer	63	0.484	1.800	0.001	0.006	0.004
hsa04935	Growth hormone synthesis, secretion and action	70	0.482	1.831	<0.001	0.003	0.002
hsa04919	Thyroid hormone signaling pathway	75	0.480	1.848	<0.001	0.004	0.002
hsa04917	Prolactin signaling pathway	40	0.479	1.619	0.011	0.034	0.020
hsa04915	Estrogen signaling pathway	67	0.475	1.784	0.001	0.006	0.004
hsa05214	Glioma	50	0.473	1.691	0.005	0.020	0.012
hsa04613	Neutrophil extracellular trap formation	75	0.472	1.816	0.001	0.006	0.004
hsa04611	Platelet activation	65	0.472	1.766	0.001	0.008	0.005
hsa04150	mTOR signaling pathway	101	0.468	1.859	<0.001	0.001	0.001
hsa04062	Chemokine signaling pathway	79	0.465	1.806	<0.001	0.005	0.003
hsa04912	GnRH signaling pathway	56	0.461	1.667	0.004	0.018	0.010
hsa05215	Prostate cancer	60	0.458	1.679	0.003	0.017	0.010
hsa00230	Purine metabolism	58	0.457	1.660	0.005	0.023	0.013
hsa04310	Wnt signaling pathway	86	0.448	1.749	<0.001	0.005	0.003
hsa01522	Endocrine resistance	60	0.442	1.620	0.008	0.030	0.017

hsa05034	Alcoholism	71	0.441	1.678	0.003	0.016	0.009
hsa04064	NF-kappa B signaling pathway	59	0.440	1.602	0.007	0.028	0.016
hsa04928	Parathyroid hormone synthesis, secretion and action	60	0.437	1.600	0.010	0.034	0.019
hsa04070	Phosphatidylinositol signaling system	64	0.436	1.621	0.002	0.014	0.008
hsa05212	Pancreatic cancer	52	0.434	1.568	0.012	0.037	0.021
hsa05211	Renal cell carcinoma	51	0.433	1.555	0.014	0.043	0.025
hsa04926	Relaxin signaling pathway	75	0.433	1.664	0.005	0.020	0.012
hsa05167	Kaposi sarcoma-associated herpesvirus infection	108	0.432	1.753	<0.001	0.004	0.002
hsa04066	HIF-1 signaling pathway	63	0.431	1.603	0.009	0.031	0.018
hsa04728	Dopaminergic synapse	71	0.431	1.641	0.004	0.019	0.011
hsa05210	Colorectal cancer	63	0.427	1.588	0.010	0.033	0.019
hsa05132	Salmonella infection	157	0.426	1.816	<0.001	<0.001	<0.001
hsa04020	Calcium signaling pathway	78	0.426	1.647	0.006	0.024	0.014
hsa04024	cAMP signaling pathway	87	0.425	1.667	0.002	0.012	0.007
hsa05200	Pathways in cancer	285	0.425	1.927	<0.001	<0.001	<0.001
hsa01240	Biosynthesis of cofactors	83	0.425	1.655	0.002	0.012	0.007
hsa04068	FoxO signaling pathway	90	0.424	1.671	0.001	0.008	0.005
hsa04210	Apoptosis	92	0.423	1.658	0.004	0.018	0.010
hsa04010	MAPK signaling pathway	170	0.423	1.816	<0.001	<0.001	<0.001
hsa03015	mRNA surveillance pathway	63	0.418	1.555	0.015	0.043	0.025
hsa04625	C-type lectin receptor signaling pathway	65	0.417	1.561	0.009	0.031	0.018
hsa05202	Transcriptional misregulation in cancer	100	0.417	1.651	0.001	0.009	0.005
hsa04722	Neurotrophin signaling pathway	73	0.416	1.586	0.010	0.033	0.019
hsa05207	Chemical carcinogenesis - receptor activation	90	0.415	1.634	0.002	0.011	0.006
hsa04933	AGE-RAGE signaling pathway in diabetic complications	64	0.409	1.520	0.009	0.031	0.018
hsa05169	Epstein-Barr virus infection	111	0.406	1.657	0.003	0.015	0.009
hsa05163	Human cytomegalovirus infection	128	0.406	1.692	<0.001	0.005	0.003
hsa05010	Alzheimer disease	209	0.405	1.778	<0.001	<0.001	<0.001
hsa05131	Shigellosis	161	0.405	1.731	<0.001	0.001	0.001
hsa04630	JAK-STAT signaling pathway	71	0.401	1.526	0.015	0.043	0.025

hsa05170	Human immunodeficiency virus 1 infection	127	0.397	1.654	0.002	0.012	0.007
hsa03013	Nucleocytoplasmic transport	77	0.397	1.532	0.016	0.046	0.027
hsa05161	Hepatitis B	109	0.395	1.602	0.004	0.017	0.010
hsa05012	Parkinson disease	140	0.393	1.648	0.001	0.006	0.003
hsa05152	Tuberculosis	85	0.388	1.523	0.009	0.031	0.018
hsa04152	AMPK signaling pathway	83	0.388	1.514	0.013	0.040	0.023
hsa04015	Rap1 signaling pathway	113	0.386	1.578	0.002	0.014	0.008
hsa04140	Autophagy - animal	101	0.384	1.527	0.008	0.031	0.018
hsa05165	Human papillomavirus infection	188	0.383	1.664	<0.001	0.002	0.001
hsa05171	Coronavirus disease - COVID-19	129	0.378	1.573	0.002	0.012	0.007
hsa05166	Human T-cell leukemia virus 1 infection	133	0.374	1.561	0.003	0.017	0.010
hsa04142	Lysosome	86	0.374	1.460	0.011	0.036	0.021
hsa05208	Chemical carcinogenesis - reactive oxygen species	120	0.367	1.515	0.009	0.031	0.018
hsa04218	Cellular senescence	109	0.364	1.479	0.013	0.040	0.023
hsa04360	Axon guidance	100	0.362	1.433	0.017	0.048	0.028
hsa04144	Endocytosis	177	0.355	1.531	0.002	0.012	0.007
hsa04141	Protein processing in endoplasmic reticulum	120	0.353	1.460	0.015	0.044	0.025
hsa05168	Herpes simplex virus 1 infection	273	0.350	1.581	<0.001	0.002	0.001
hsa04151	PI3K-Akt signaling pathway	177	0.337	1.453	0.007	0.026	0.015
hsa05022	Pathways of neurodegeneration - multiple diseases	246	0.329	1.472	0.001	0.008	0.005

$P < 0.05$ was considered statistically significant.

Table S5. GSEA enrichment analysis at mRNA levels

ID	Description	Set size	Enrichment score	NES	P-value	P.adjust	q-value
hsa05168	Herpes simplex virus 1 infection	272	-0.385	-1.897	0.000	0.000	0.000
hsa05166	Human T-cell leukemia virus 1 infection	133	-0.400	-1.793	0.000	0.023	0.019
hsa04380	Osteoclast differentiation	67	-0.465	-1.813	0.001	0.032	0.027
hsa04064	NF-kappa B signaling pathway	57	-0.471	-1.804	0.001	0.043	0.036
hsa04668	TNF signaling pathway	76	-0.473	-1.921	0.000	0.020	0.017
hsa05210	Colorectal cancer	63	-0.475	-1.845	0.001	0.049	0.042
hsa04625	C-type lectin receptor signaling pathway	65	-0.487	-1.893	0.001	0.032	0.027
hsa04630	JAK-STAT signaling pathway	70	-0.500	-1.979	0.000	0.020	0.017
hsa04657	IL-17 signaling pathway	43	-0.521	-1.892	0.001	0.032	0.027

$P < 0.05$ was considered statistically significant.

Table S6. Antibodies used in experiments

Antibodies	Source	Application	Identifier
Anti-CD63	CST	WB	52090S
Anti-Alix	CST	WB	92880
Anti-TSG101	CST	WB	72312
Anti-GM130	CST	WB	12480
Anti-CD68	Abcam	IF	ab213363
Anti-CD4	CST	IF	93518S
Anti-CD8α	CST	IF	55397
Anti-α-SMA	CST	IF	19245
Anti-Foxp3	R&D	IF	MAB8214
Anti-iNOS	Abcam	IF	ab178945
Anti-CD163	Abcam	IF	ab182422
Anti-CD206	CST	IHC	24595
Anti-ALKBH5	Proteintech	WB; IF; RIP	16837-1-AP
Anti-GAPDH	Proteintech	WB	60004-1-Ig
Anti- Histone H3	Abcam	WB	ab1791
Anti-p-JAK2	Abcam	WB; IHC	ab32101
Anti-JAK2	Abcam	WB	ab108596
Anti-p-STAT5	CST	WB; IHC	9359
Anti-STAT5	CST	WB	94205
Anti-SOCS2	Sigma	WB; IHC	SAB5701688
Anti-Ki67	Abcam	IHC	ab16667
Anti-METTL3	Abcam	RIP	ab195352
Anti-METTL14	Proteintech	RIP	26158-1-AP
Anti-WTAP	Abcam	RIP	ab195380
Anti-FTO	Proteintech	RIP	27226-1-AP
Anti-N6-Methyladenosine	Synaptic Systems	Dot Blot; RIP	202003

Table S7. Primer Sequences

Primer name	Forward (5' - 3')	Reverse (5' - 3')
hsa_circ_0008253	*ACTTCTTGCCTGCTCTCAGTT*	*CTCTGGTCTCTCTTCTCGGG*
hsa_circ_0004606	*TGCAGAACTATGTCCTCACCC*	*GGTACACCACATGTGTCCTC*
hsa_circ_0017639	*TTGGGATGAAGCTTGAGACA*	*CTCGAACCAGTCAAGTCACG*
hsa_circ_0003314	*CCTCTTCTGCCTCGTCATGG*	*CCACTTGCCAGGGAAAATGAT*
hsa_circ_0077086	*GCTTTAAGCTGCCAAAGTTCCT*	*TTCAACACCTTGGAGCCGAC*
hsa_circ_0040148	*GTCTGAGTTGTCCTATGCCCC*	*AGCAGGTTGCTGTCTTTCGG*
hsa_circ_0042521	*ATGAAGCTGTTCATCTCCTTTGC*	*CTGGCTAGACAGGGTATAGAGT*
hsa_circ_0034447	*GAGCTGTCCAGCATGGTCCT*	*GTGGCCAATGTAGTTAGTGCG*
hsa_circ_0072758	*CAGGTTGTTGCAGGAGACCAT*	*AGAGAAAGCCTGACACAGGTC*
hsa_circ_0002360	*ACCACTCCACTGCCTTTAACC*	*GGGGCCCATCCACTGTGATT*
hsa_circ_0054241	*ATGGGACTGGCAGAAGAAAGC*	*CACGCTCAAAAGTGCCAAGT*
hsa_circ_0003528	*GGTTCCTTTTTCTTTGCGGCT*	*TGCTGACCAGAACAGTCCAAG*
circEML4	*AACACAGGCTGGAATGGACC*	*CGCTCAAAAGTGCCAAGTCC*
circEML4 convergent	*TGTGGGATTCTGTTACTCTA*	*TATGACCTCTGCTCCTTTT*
GAPDH	*CAATGACCCCTTCATTGACC*	*TTGATTTTGGAGGGATCTCG*
GAPDH divergent	*TCAAGAAGGTGGTGAAGCAGGC*	*ATGCCAGTGAGCTTCCCGTT*
EML4	*GACATTGATTGGACGACAT*	*TGTACTTGTGACTGGGAGC*
SOCS2	*TGCAAGGATAAGCGGACAGG*	*CAGAGATGGTGCTGACGTGT*
CCL-22	*AAACTAATGTCCCTCCCCTCTC*	*TTTGGGGCTTCACATTGACC*
TGF-β	*CAACAATTCCTGGCGATACC*	*GAACCCGTTGATGTCCACTT*

Table S8. Sequences of siRNAs

Name	Sequence
circEML4 siRNA-1	*ACGCACTCAGGCAGCCTCT*
circEML4 siRNA-2	*CACTCAGGCAGCCTCTACA*
circEML4 siRNA-3	*TCAGGCAGCCTCTACAACC*
ALKBH5 siRNA-1	*GATCGCCTGTCAGGAAACA*
ALKBH5 siRNA-2	*GTCCTTCTTTAGCGACTCT*
ALKBH5 siRNA-3	*GCTGCAAGTTCCAGTTCAA*

Table S9. Sequences for FISH

Name	Sequence
5-FAM-labeled circEML4_probe	*TAGAGGC+TGCCTGAGTGCGT+TCCTA*
Cy3-labeled circEML4_probe	*GT+TGTAGAGGCTGCC+TGAGTGCGT*

Table S10. Sequences for RNA pull-down assay

Name	Sequence
circEML4_probe-1	*TGGGGTTGTAGAGGCTGCCTGAGTG*-/3bio/
circEML4_probe-2	*TGTAGAGGCTGCCTGAGTGCGTTCC*-/3bio/
NC probe	*AAGTGCTGGTGTTGCCGGGGTGTTC*-/3bio/
